# Magnetic hysteresis experiments performed on quantum annealers

**DOI:** 10.1126/sciadv.aeb5192

**Published:** 2026-02-27

**Authors:** Elijah Pelofske, Frank Barrows, Pratik Sathe, Cristiano Nisoli

**Affiliations:** ^1^ Information Systems and Modeling, Los Alamos National Laboratory, Los Alamos, NM, USA.; ^2^ Theoretical Division, Quantum and Condensed Matter Physics, Los Alamos National Laboratory, Los Alamos, NM, USA.; ^3^ Center for Nonlinear Studies, Los Alamos National Laboratory, Los Alamos, NM, USA.; ^4^ Information Science and Technology Institute, Los Alamos National Laboratory, Los Alamos, NM, USA.

## Abstract

While quantum annealers have emerged as versatile and controllable platforms for experimenting on correlated spin systems, the important phenomenology of magnetic memory and hysteresis remain unexplored on hardware designed to escape metastable states via quantum tunneling. Here, we present the first general protocol to experiment on magnetic hysteresis on programmable quantum annealers and implement it on three D-Wave superconducting qubit quantum annealers, using up to thousands of spins, for both ferromagnetic and disordered Ising models, and across different graph topologies. We observe hysteresis loops whose area depends nonmonotonically on quantum fluctuations, exhibiting both expected and unexpected features, such as disorder-induced steps and nonmonotonicities. Our work establishes quantum annealers as a platform for probing nonequilibrium emergent magnetic phenomena, thereby broadening the role of analog quantum computers into foundational questions in condensed matter physics.

## Teaser

A programmable magnetic hysteresis experiment protocol on analog quantum computers is introduced.

## INTRODUCTION

Complex models of frustrated magnets (*1*–*7*) and topological spin liquids (*6*, *8*, *9*) have recently been realized on analog quantum annealers (*10*–*12*), increasingly used as programmable dynamical laboratories with controllable quantum fluctuations and direct access to individual spin configurations. Nevertheless, a gap remains. Systematic studies of hysteresis, the landmark feature of magnetic systems, are still lacking, limiting our understanding of out-of-equilibrium, memory-preserving dynamics under external fields. Although some works have explored field-induced phases (*9*, *13*)—notably, one study on out-of-equilibrium magnetization response that inspired the present work (*13*)—these were context specific, did not provide a general framework for magnetic hysteresis, and did not demonstrate memory retention through a full cycle of longitudinal field sweeps. The challenge stems from the hardware design. Annealing protocols exploit quantum tunneling to escape local minima for combinatorial optimization (*14*), inherently erasing path-dependent memory. This conflicts with hysteresis, which relies on memory retention and metastability. Nonetheless, a controlled activation of the transverse field has been shown to retain a disordered spin configuration while “kicking” topological defects (*6*, *9*, *15*).

We demonstrate quantum annealers as experimental platforms to perform hysteresis measures on collective spin systems. We apply a time-varying longitudinal field to a system of interacting qubits while maintaining quantum fluctuations that facilitate transitions and show that the system retains memory: The outcomes are explicitly dependent on the history of the applied field, resulting in hysteresis. Memory is tunable and varies nonmonotonically with the magnitude of the transverse field, which sets the strength of quantum fluctuations.

We showcase our methodology on three representative models across three different machines. In simple high-coordination ferromagnets, we observe the expected box-shaped hysteresis. In systems of disordered coupling, we observe Barkhausen noise, also as expected. In lower-coordination, disordered systems, we detect intriguing pinched nonmonotonicities that have been previously observed in certain spin glasses and whose mechanism remains poorly understood.

Our approach is not merely a simulation but an experiment on superconducting qubits, conceptually identical to techniques used to probe magnetic materials in a laboratory. As such, it not only offers advantages over conventional simulations, which typically require clever algorithms with nonequilibrium computations to approximate kinetics (*16*) that are often tailored to the specific system under study, but also represents an epistemologically distinct approach.

## RESULTS

We use three D-Wave quantum processing units (QPUs) on the basis of superconducting flux qubits (*17*–*19*), interconnected by couplers arranged in machine-specific graphs known as Pegasus (*20*, *21*) and Zephyr (*22*). The chip IDs of three QPUs are Advantage_system4.1 (5267 qubits), Advantage_system6.4 (5612 qubits), and Advantage2_prototype2.6 (1248 qubits). They implement the time-dependent transverse-field Ising Hamiltonian (*23*)

$$H=-\Gamma \sum_{i}{\hat{\sigma }}_{x}^{i}+\sum_{i}h_{i}{\hat{\sigma }}_{z}^{i}+\sum_{\langle \mathrm{ij}\rangle }J_{\mathrm{ij}}{\hat{\sigma }}_{z}^{i}{\hat{\sigma }}_{z}^{j}$$ (1)

where ${\hat{\sigma }}^{i}$ are Pauli matrices at node *i* and $J_{\mathrm{ij}}$ are couplings along edges 〈ij〉.

When Γ = 0, the “longitudinal” Hamiltonian is diagonal in the ${\hat{\sigma }}_{z}$ basis. For Γ ≠ 0, the transverse term does not commute with ${\hat{\sigma }}_{z}$, introducing quantum fluctuations that drive transitions among states of the longitudinal Hamiltonian. An Ising spin model is programmed on D-Wave hardware by specifying the interaction values $J_{\mathrm{ij}}$ for each coupler and $h_{i}$ for each qubit.

In standard quantum annealing, the energy scale $J_{\mathrm{ij}}$ increases while Γ reduces to zero, driving the system from a quantum paramagnetic state toward a “classical” configuration by progressively suppressing quantum fluctuations. Specifically, in D-Wave quantum annealer, the Hamiltonian above takes the form

$$\begin{array}{cc} H= & -\frac{A\left(s\right)}{2}\sum_{i}{\hat{\sigma }}_{x}^{i}\\ & +\frac{B\left(s\right)}{2}\left(g\left(t\right)\sum_{i}{\tilde{h}}_{i}{\hat{\sigma }}_{z}^{i}+\sum_{\langle \mathrm{ij}\rangle }{\tilde{J}}_{\mathrm{ij}}{\hat{\sigma }}_{z}^{i}{\hat{\sigma }}_{z}^{j}\right) \end{array}$$ (2)

*A*(*s*) and *B*(*s*) are hardware-specific functions, decreasing and increasing respectively in the annealing parameter *s* that varies in time from 0 to 1 (see Supplementary Information G). Critically, the global time-dependent multiplier *g*(*t*) (or “h-gain”) scales all local fields and can be varied independently from *s*(*t*): This hardware feature is central to our experiments.

### Protocol

**Fig. 1.**
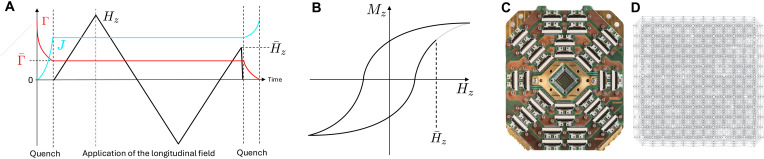
Schematic magnetic hysteresis protocol and D-Wave QPU. (**A**) Schematic showing the time dependence of Γ, *J*, and $H_{z}$ in Eq. 1, used to generate the magnetization at field ${\bar{H}}_{z}$. Initially, the annealing parameter *s* is rapidly (0.5 μs) increased to a fixed value, setting Γ and *J* to their target values. Γ (red) and *J* (cyan) remain fixed while the longitudinal full sweep of $H_{z}$ traces the conceptual schematic hysteresis loop shown in (**B**), with readout occurring at many intermediate points denoted as ${\bar{H}}_{z}$ at simulation times after the dashed vertical purple line. A final quench of Γ and *J*, also lasting 0.5 μs, allows final readout in the *Z* basis. Before that, we rapidly quench the longitudinal field *H* in 20 ns to stop the longitudinal field from being applied while the quench in Γ and *J* occurs; field sweeps span up to 10 μs (see Supplementary Information A for more implementation details). (**C**) A Pegasus D-Wave QPU chip and (**D**) the native graph architecture of Advantage2_prototype2.6, one of three quantum annealing processors used in this study.

Our protocol, illustrated in Fig. 1, begins by rapidly quenching the annealing parameter *s* from 0 to a target value in the range *s* = 0 to 1, within ~0.5 μs, setting the transverse field Γ and the coupling strength *J*. Holding *s* (and, thus, *J* and Γ) fixed in time, we apply a (spatially) uniform longitudinal field $h_{i}=H_{z}$ through various values of the loop by programming the global scaling function *g*(*t*). The sweeps, possibly positive and negative, stop at a field ${\bar{H}}_{z}$, after which *g*(*t*) is quickly (in ≈20 ns) brought to zero and *s* is ramped to 1, quenching Γ (in 0.5 μs) before the qubits are measured in the ${\hat{\sigma }}_{z}$ basis, from which the average magnetization $M_{z}$, as well as other observables, can be extracted by repeated hardware anneal-readout cycles (in this study, we measure 2000 samples for each ${\bar{H}}_{z}$). This cycle is repeated across a range of ${\bar{H}}_{z}$ values to reconstruct the full probe-response dynamics.

Each Ising spin maps to a physical qubit on the hardware lattice. Autoscaling of programmed Ising model coefficients is disabled to preserve intended energy scales. The D-Wave QPUs operate at ~15 mK, and experiment timescales exceed closed quantum system decoherence times, which are estimated to be ~100 ns at most (*24*–*28*), placing the dynamics in a quasistatic, noisy, open-system regime (*29*–*31*). All experiments in this study use a total simulation time, e.g., annealing time, of 11.2 μs (see Supplementary Information A).

### Experiments

We test our hysteresis protocol on three distinct types of Ising models and demonstrate hysteresis and memory. We begin with the ferromagnet on the full hardware graph, a natural test case. The Pegasus graph D-Wave processors have an average coordination number of ~14. See also table S1 in Supplementary Information A for precise details regarding qubit connectivity. Results for Advantage_system4.1, which are nearly identical, are shown in Supplementary Information B.

**Fig. 2.**
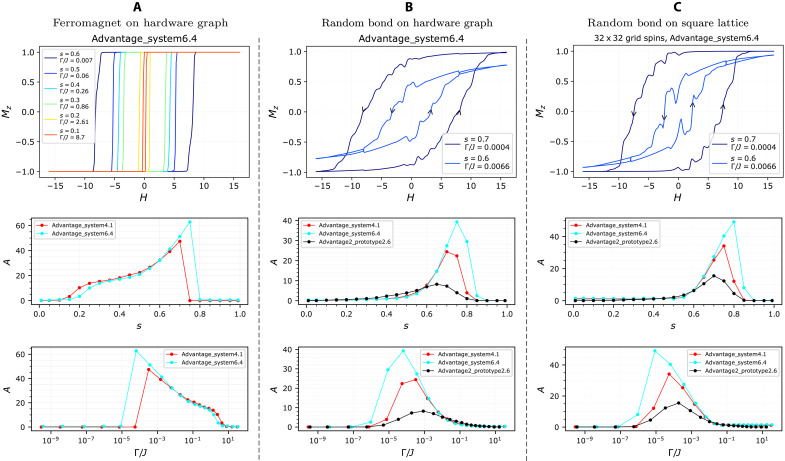
Magnetic hysteresis experimental results on D-Wave QPUs. The three columns correspond to (**A**) ferromagnet on the hardware graph, (**B**) binary random bond (±*J*) model on the hardware graph, and (**C**) binary random bond (±*J*) model on a square lattice (with open boundary conditions). Top row: Plots of normalized magnetization $M_{z}$ versus longitudinal field *H*, for the three models, all implemented on the same Advantage_system6.4 processor (see Figs. 3 and 4 for results across all three devices) exhibit closed-loop magnetic hysteresis. The arrows along the magnetization curves denote simultaneously the direction of the longitudinal field sweep and the time progression of the protocol. The ferromagnet loops exhibit a “boxed” shape indicative of high coordination and mean-field behavior; the *±J* models show crackling noise and nonmonotonic features expected from structural disorder. Middle row: Hysteresis loop area versus hardware annealing parameter *s* for three different D-Wave QPUs. Bottom row: Hysteresis loop area as a function of equivalent physical ratio Γ/*J* to the *s* value plots shown in the middle row.

Figure 2A shows average magnetization per spin versus longitudinal field on Advantage_system6.4, revealing clear hysteresis and memory. The rectangular shape is consistent with the mean-field behavior expected in ferromagnets of high coordination, although tiny deviations are visible in the case of extremely small quantum fluctuations (*s* = 0.6), a regime in which the graph’s discrete topology is less washed out.

Our second and third models exhibit considerably more complex behavior. We realize a binary random bond model on the full hardware graph and also on a square lattice with open boundary conditions. In the latter case, we embed the square lattice using a fixed hardware-native subgraph isomorphism using the Glasgow Subgraph Solver (*32*), embedding square grids of size 32 × 32 on Advantage_system4.1 and Advantage_system6.4 and size 26 × 26 on Advantage2_prototype2.6. In both cases, all couplings have fixed magnitude *J* but random sign (±*J*) in equal ratio, a coexistence of ferromagnetic and antiferromagnetic interactions known to lead to frustration and glassy behavior (*33*, *34*). While full spin glass characterization is beyond the scope of the current study, here we highlight the potential of our method by showing that the introduction of disorder leads to considerably more complex hysteresis. In particular, it should give rise to Barkhausen noise, which is a hallmark of disordered magnetic systems (*35*–*39*).

Figure 2 (B and C) reports considerably more complex hysteresis loops for these two random bond cases. We observe the corrugations and crackling noise, suggestive of discrete avalanche-like spin reconfigurations, typical of the disorder-induced, jerky slipping of magnetic domains, whose potential fractal structure could be investigated in future works.

**Fig. 3.**
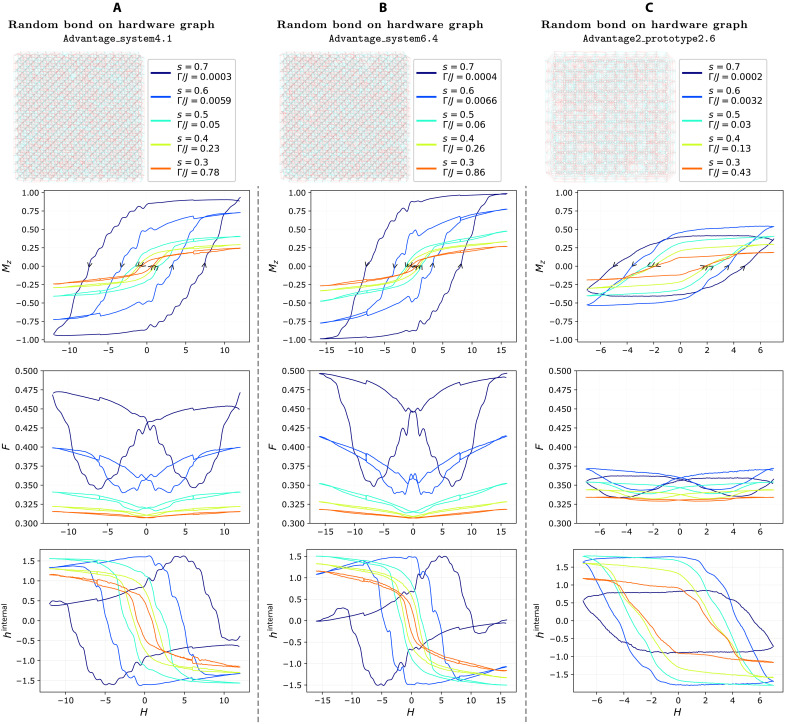
Magnetic hysteresis for the ±*J* model on full hardware graphs. The three distinct hardware-defined ±*J* models are shown on top (*J* = −1 edge coupling is cyan, and *J* = +1 is red) on (**A**) Advantage_system4.1, (**B**) Advantage_system6.4, and (**C**) Advantage2_prototype2.6. In the rows below, we present average magnetization $M_{z}$, fraction of frustrated bonds F, and internal field $h^{\text{internal}}$, plotted against the longitudinal field *H* for various values of *s* (and thus different Γ/*J* ratios) specified in the legends of the top row. For sufficiently large Γ/*J*, the hysteresis loop area almost disappears as the model approaches a quantum paramagnet. The arrows along the magnetization curves simultaneously denote the direction of the longitudinal field sweep and the time progression of the protocol. Note that Advantage2_prototype2.6 (C) does not fully magnetize.

**Fig. 4.**
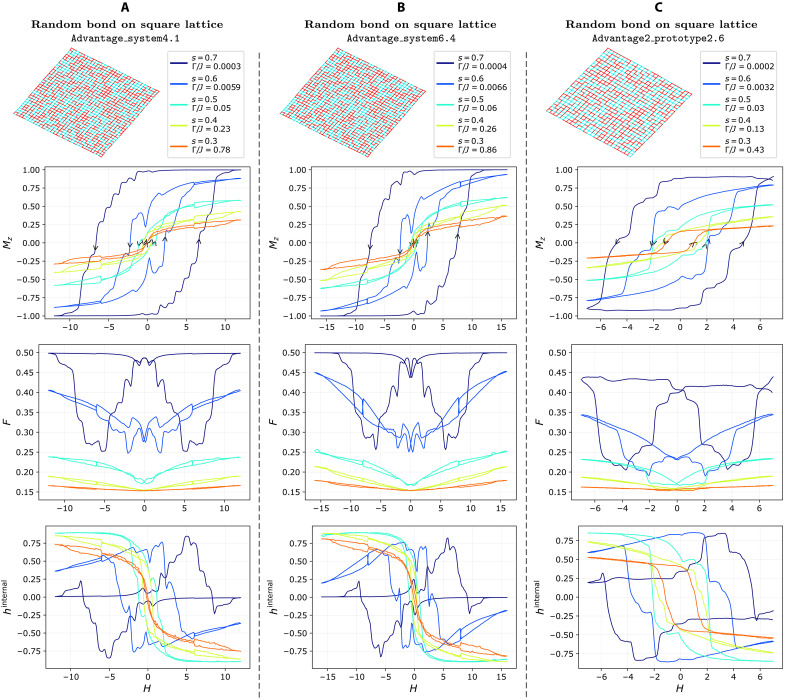
Magnetic hysteresis for the ±*J* model defined on 2D square lattices. The three distinct ±*J* models on a square graph, with open boundary conditions, are shown on top row (*J* = −1 is red and *J* = 1 is cyan), corresponding to the three different D-Wave processors. The same 32 × 32 instance is run on both Advantage_system4.1 (**A**) and Advantage_system6.4 (**B**), while a 26 × 26 ±*J* model is run on Advantage2_prototype2.6 (**C**). In the following rows, we present magnetization $M_{z}$, fraction of frustrated bonds F, and internal field $h^{\text{internal}}$, plotted against the applied field for various values of *s* (and, thus, different Γ/*J* ratios) specified in the legends of the top row. For sufficiently large Γ/*J*, the hysteresis area almost disappears as the model approaches a quantum paramagnet. Unlike in Fig. 3, the lower coordination of the Ising model allows for full saturation, where as predicted F=0.5 and $h^{\text{internal}}=0$ in column (A) and (B), but not in (C), due to the lower maximum achievable longitudinal field on the Advantage2_prototype2.6 processor.

We also observe intriguing nonmonotonicities in the curves, particularly visible for the square lattice in panel (C), where strong dips emerge. This phenomenon has been previously reported, e.g., in Ge_0.87_Mn_0.13_Te spin glasses (*40*, *41*), where is not yet understood, as well as in the competition between internal and collective magnetization of nanolements (*42*). As a working hypothesis, to be tested in future work, we speculate that, in our ±*J* model, competition between ferro- and antiferromagnetic couplings can induce local spin rearrangements, such as reentrant ferromagnetic states. The effect is enhanced at intermediate transverse fields, where quantum fluctuations reduce the area of hysteresis but amplify dips and corrugations (see Figs. 3 and 4), suggesting that tunneling between nearly degenerate states of different magnetization plays a role. In future work, a comparison of these results and classical simulations might clarify the role, if any, of quantum tunneling.

The memory content can be quantified as the area enclosed by the hysteresis loop and is expected to vary nonmonotonically with the ratio Γ/*J*: At high Γ/*J*, quantum fluctuations should suppress memory as the system approaches quantum paramagnetism; instead, at very low Γ/*J*, spins should remain unresponsive. Figure 2 shows the hysteresis area as a function of the annealing parameter *s* and the corresponding Γ/*J* ratio across different machines, confirming this nonmonotonic behavior. In the ferromagnetic case, the profiles are nearly identical across the two devices, despite slight differences in the hardware graphs, reflecting the mean-field behavior already mentioned. In contrast, the random bond models show substantial variation in hysteresis area at small Γ/*J*. This is expected in the full-graph case because the hardware graphs differ across machines and also because different machines are more or less able to reach full polarization. Machine-intrinsic timescale variations may also affect the hysteresis loop area.

A more complete picture is afforded by Figs. 3 and 4, reporting data for the binary random bond models, implemented respectively on the full hardware graph and on the square lattice embedding with open boundary conditions, across the three quantum annealing devices. For the full hardware graph case, full magnetic saturation is not reached (Fig. 3, second row), although Advantage_system6.4 approaches it closely because of its stronger *h*-gain capability (see Supplementary Information A). On Advantage2_prototype2.6, high average connectivity (average coordination number of ≈17) and limited maximum longitudinal field strength prevent $M_{z}$ from exceeding 0.5, resulting in minor hysteresis loops, which are generally characterized by smoother profiles because of more localized magnetic responses (*43*, *44*).

In the square lattice case (Fig. 4, second row), full magnetization is achieved on both Advantage_system4.1 and Advantage_system6.4. Even at relatively high transverse-to-coupling ratios (Γ/*J* = 0.78 and 0.86, respectively), the system never really approaches the quantum paramagnetic regime, and an intriguing pinched hysteresis appears, more typical of memristors. While vanishing magnetization at zero field is expected under strong quantum fluctuations, the persistence of some history-dependent behavior in this regime highlights the robustness of the memory effect, even as quantum tunneling dominates. Note that Fig. 4 (A and B) shows experiments with the same random distribution of couplings, leading to nearly identical results across the two devices. The small differences are due to the fact that equal *s* values correspond to slightly different Γ and *J* absolute values. Here, too, on Advantage2_prototype2.6, magnetization never fully saturates, resulting in minor loops.

We stress here the importance of enabling systematic exploration of minor loops. These are inaccessible to previous ad hoc approaches (*13*) yet essential to enabling experimental studies of return-point memory, training effects, and the emergence of limit cycles in driven spin systems, as we will report in future work.

### Microscopic vistas

The results presented, so far, resemble those obtainable in a laboratory setting, but quantum annealers offer unique access to individual spin-level resolution for the extraction of microscopic quantifiers. We highlight a few examples to illustrate the broader potential of our methodology. For a useful quantifier, we can define the average frustration F, as the fraction of frustrated couplings, or

$$F=\langle \frac{1}{2N_{e}}\sum_{\langle \mathrm{ij}\rangle }\left[1+\text{sgn}\left(J_{\mathrm{ij}}\right)\sigma_{z}^{i}\sigma_{z}^{j}\right]\rangle$$ (3)

where sgn is the sign function, 〈⋅〉 denotes a sampling average, and $N_{e}$ is the total number of couplers, and the sum is over all qubits.

We can also extract internal “forces,” such as the effective internal fields acting on each spin

$$h_{i}^{\text{internal}}=-\sum_{j\in {\text{∂}}_{i}}J_{\mathrm{ij}}\sigma_{z}^{j}$$ (4)

where ${\text{∂}}_{i}$ denotes the nodes coupled to site *i*. From it, the average internal field is $h^{\text{internal}}=\langle \frac{1}{N_{s}}\sum_{i}\;h_{i}^{\text{internal}}\rangle$

In our ±*J* model, F=1/2 and $h^{\text{internal}}=0$ at saturation because of the equal number of ferromagnetic and antiferromagnetic couplings. The third rows of Figs. 3 and 4 show “butterfly”-shaped hysteresis curves for the frustration F. In both models, we see that F reaches the expected maximum of 0.5 only where magnetization saturates to 1, as predicted. At equilibrium, configurations at zero field minimize frustration, but this is generally not the case in hysteretic, out-of-equilibrium regime. However, both figures show that are larger Γ/*J*, the system achieves considerably lower frustration, consistent with a more effective annealing from stronger quantum fluctuations.

Similarly, the fourth row of Figs. 3 and 4 plots the average internal field $h^{\text{internal}}$ versus $H_{z}$, which vanishes at full saturation, as predicted. Notably, the hysteresis structure of $h^{\text{internal}}$ is more intricate than that of magnetization, displaying multiple pinch points. More generally, out-of-equilibrium anomalies such as nonmonotonicities, asymmetries, switches, and memory are more pronounced in F and $h^{\text{internal}}$ than in $M_{z}$.

In the full-graph case (Fig. 3), minimal frustration occurs near zero magnetization. In the hysteretic regime, this aligns with the coercive field, while, in the quantum paramagnetic regime (large Γ/*J*), it instead coincides with zero field, where the butterfly collapses. In contrast, the square lattice case (Fig. 4) shows minimal frustration before coercivity, and it aligns with the maximum absolute value of $h^{\text{internal}}$.

Full saturation erases any memory of prior states, resulting in central symmetry (with respect to the origin) of the hysteresis curve. Instead, when the magnetization does not fully saturate, for minor loops, this symmetry can be lost. This effect is evident in Fig. 4A, for *s* = 0.7, and in Fig. 3C, also for *s* = 0.7, both of which exhibit a breakdown of central symmetry in their hysteresis loops. This asymmetry becomes more pronounced in the corresponding curves for F.

**Fig. 5.**
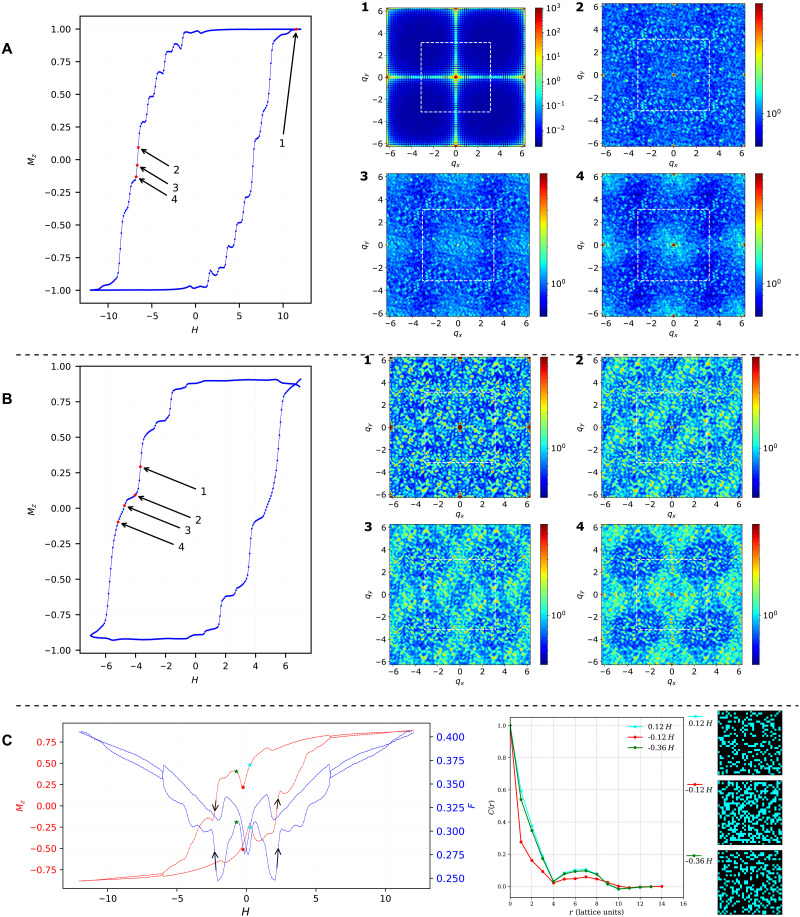
MSFs and real-space configurations. Hysteresis curves at *s* = 0.7 and selected MSFs for (**A**) the 32 × 32 ±*J* run on Advantage_system4.1 and (**B**) the 26 × 26 ±*J* run on Advantage2_prototype2.6. All MSF plots use a log scale S(q) heatmap, and the dashed white box outlines the first Brillouin zone. Panel (**C**) highlights an nonmonotonic dip in magnetization by showing $M_{z}$ and coupler frustration hysteresis F data overlaid, from a 32 × 32 ±*J* model run on Advantage_system4.1 at *s* = 0.6. Panel (C) also reports correlations along principal axes (line colors correspond to the annotated colors along the hysteresis loop), and example single-sample real-space spin configurations, with black (cyan) pixels corresponding to −1 (+1) spins, from before, at, and after, the nonmonotonic magnetization dip.

By providing access to measured spin configurations, our methodology allows also extraction of magnetic structure factors (MSFs)

$$S\left(q\right)=\langle \sum_{i,j}e^{\mathrm{iq}\cdot \left(r_{i}-r_{j}\right)}\sigma_{z}^{i}\sigma_{z}^{j}\rangle$$

(*r* is measured in units of the lattice constant, and the average is more than 100 sampled spin configurations). Figure 5 (A and B) shows MSFs at different points of the hysteresis loops for the random bond model on a square lattice at *s* = 0.7, across two machines. For improved visualization clarity, in Fig. 5, MSF plots used capped ∣S(q)∣ when plotting the heatmaps. Specifically, for all MSFs in panel (A), ∣S(q)∣ was capped at 8, and, for panel (B), they were capped at ∣S(q)∣=6. Panel (A) tells a familiar story of the fading of the central Bragg peak (at MSF 1), typical of the saturated state that the central Bragg peak dominates (the surrounding weaker peaks on the axes, more apparent due to the logarithmic scale, are a finite size effect) as the system demagnetizes (MSFs 2 to 4), leading to the diffuse intensity of frustrated disorder, with residual short range ferromagnetic correlations.

Figure 5B reports more distant points along the hysteresis loop, with increased structure in the demagnetized state. At MSF 1, intensity at the Brillouin zone boundary suggests emerging antiferromagnetic correlations atop weak ferromagnetic order, whereas, at MSFs 2 to 4, fourfold symmetry breaking reflecting the disordered coupling, low edge intensity, and absent corner peaks suggests stripe-like rather than Néel antiferromagnetic order.

Figure 5C examines three points around a dip, labeled cyan, red, and green. While magnetization decreases from cyan to red but then unexpectedly increases from red to green. *C*(*r*), the correlation function along the *x* and *y* axes, whose undulated shape suggests a “liquid” of ferromagnetic domains, is effectively identical before and after the dip, suggesting reentrant ferromagnetic domains. This speculation is corroborated by real-space spin maps revealing larger ferromagnetic domains at these points. This reentrance is likely of quantum origin: The fraction of frustrated energy links F, plotted in red on top of the magnetization curve, also shows a corresponding dip, signifying that the “internal energy,” like the longitudinal Zeeman energy, is reduced at the dip, but both increase as the longitudinal field is further reduced. The only possible energy gain to justify such a reentrant phase would be coming from the transverse Zeeman energy, the first term in Eq. 1. Due to the experimental unavailability of projective measurements in the *x* basis, our hypothesis cannot now be tested. Nonetheless, our results demonstrate how microscopic access can provide working hypotheses to be explored in future work or with more advanced quantum processor capabilities. Supplementary Information D examines this nonmonotonic dip using averaged MSFs.

## DISCUSSION

We have demonstrated that quantum annealers are viable platforms for the study of memory and hysteresis and that their memory can be finely tune by controlling quantum fluctuations. Our approach, particularized for D-Wave analog quantum computers, closely mimics laboratory protocols but with the added, crucial advantage of a direct sampling of individual spin configurations, allowing for spatially resolved measurements of magnetization, internal fields, frustration, correlations, structure factors, and other essential measures for probing disordered and glassy systems, where bulk observables often obscure local dynamics and emergent behavior. We have showcased the rich phenomenology of our results to illustrate the broader potential of our method, although we leave to future works the full explanation of its many intricacies.

Our results are transferable across different quantum annealing processor hardware platforms when the model is identical, with deviations appearing mainly at very small Γ/*J* or due to limited longitudinal field strength. For instance, Advantage2_prototype2.6 cannot fully polarize a square lattice with ~50% antiferromagnetic bonds, rendering it unsuitable for such studies. Polarization would be possible at a reduced *J* that would, however, raise the effective temperature and further hide possible quantum effects. Current limitations in the programmable longitudinal field schedule (see Supplementary Information A) hinder the exploration of further experiments such as training and cycles. Future quantum processors may also support faster quenches and higher qubit coherence, which would enable more precise studies of quantum behavior.

Our methodology enables direct experimental access to corrugated and noisy profiles, typical of disordered systems, and opens a path toward future experimental studies of their potentially fractal scaling in a tunable quantum system, as well as the investigation of training effects and limit cycles in hysteresis, phenomena central to Preisach-type models that extend beyond magnetism. In future works, we will explore precise probing of minor loops, return-point memory, ergodicity breaking, and spin glass aging, all characterized at the level of individual degrees of freedom, capabilities not accessible through previous approaches. Moreover, the sweep rate of the external field can be tuned, enabling controlled exploration of timescale effects in out-of-equilibrium dynamics (see Supplementary Information A), and the longitudinal field could optionally be held constant during the final quench.

Beyond magnetism, the ability to engineer path-dependent responses has implications for neuromorphic computing, adaptive memory materials, artificial synapses, and associative memory models. More broadly, our results establish quantum annealers as experimental testbeds for nonequilibrium statistical mechanics, offering access to dynamical phenomena that are otherwise inaccessible to classical simulations or condensed matter experiments. By reimagining quantum hardware as fully programmable laboratories for hysteresis and memory, this work sets a benchmark for the scientific utility of quantum annealers and lays the groundwork for a previously unidentified class of quantum computer enabled experiments.

## MATERIALS AND METHODS

While some of the methodology has already been discussed in the “Protocol” section, we provide a brief summary of it here. For more details, we refer the reader to the Supplementary Information.

Experiments were performed on three D-Wave QPUs: Advantage_system4.1 and Advantage_system6.4 (both with Pegasus topology) and Advantage2_prototype2.6 (Zephyr topology). They differ in energy scales and maximum programmable longitudinal fields (see Supplementary Information A). All devices implement the transverse-field Ising Hamiltonian of Eq. 2, with programmable couplers $J_{\mathrm{ij}}$ and local fields $h_{i}$. Each hysteresis experiment is a probabilistic sampling based protocol that uses modified “anneals” in which the system is held at a fixed anneal fraction *s* (typically in the range 0.3 to 0.7), which sets the ratio Γ/*J* (e.g., *s*). While keeping *s* fixed, a spatially uniform longitudinal field $H_{z}$ is applied in a time-periodic manner using the programmable function *g*(*t*), via the “h-gain schedule” functionality. The magnetic state of the qubits cannot be continuously monitored during the course of the evolution because the underlying hardware is an analog quantum computer, and, hence, data are collected by repeating anneal-readout cycles at discrete $H_{z}$ values. The hardware control necessary to carry out this experiment is time-dependent longitudinal fields and time-dependent control of Γ/*J*. The Ising models that we consider are disordered and frustrated ±*J* models, as well as pure hardware-defined ferromagnets, and they are mapped to the hardware by direct spin to qubit embedding. Each full loop uses a total (final) simulation time of 11.2 μs, with ~500 linearly spaced field points (time resolution, 0.01 μs) and 2000 independent samples per point. The ramp to and from the paused *s* value lasts 0.5 μs; quenches of the longitudinal field before final ramp are considerably faster and last 20 ns. A 100-ns pause is inserted before and after each sweep segment. Magnetization $M_{z}$ is computed by averaging over qubits and samples. The magnetic spin structure factor S(q) is computed on a 200 × 200 grid and averaged over 100 sampled spin configurations.

## Supplementary Materials

### This PDF file includes:

Supplementary Information A to H Figs. S1 to S10 Table S1 References
