# Supplementary material for: Magnetic hysteresis experiments performed on quantum annealers
Source: Sci Adv. 2026 Feb 27;12(9):eaeb5192. doi: 10.1126/sciadv.aeb5192 (PMC12947867; doi:10.1126/sciadv.aeb5192)
Supplement: Supplementary file 1 — Supplementary Information A to H Figs. S1 to S10 Table S1 References [file sciadv.aeb5192_sm.pdf]

Science Advances
AAAS

Supplementary Materials for

Magnetic hysteresis experiments performed on quantum annealers

Elijah Pelofske *et al.*

Corresponding author: Elijah Pelofske, epelofske@lanl.gov; Frank Barrows, fbarrows@lanl.gov; Pratik Sathe, psathe@dwavesys.com; Cristiano Nisoli, cristiano@lanl.gov

Sci. Adv. **12**, eaeb5192 (2026)
DOI: 10.1126/sciadv.aeb5192

This PDF file includes:

Supplementary Information A to H
Figs. S1 to S10
Table S1
References

Supplementary Information A: Magnetic Hysteresis Protocol and Details of the Machines

For details about the three QPUs used in this study, see Table S1. Before going into the specific details of our protocol, we further contextualize our work in the broader context of the annealing processes implemented on D-Wave's QPUs.

The time-dependent Hamiltonian implemented on D-Wave's QPUs is given by Eq. (2). Quantum annealing typically proceeds as follows. All active qubits are initialized to the all up state in the Pauli-x basis. Next, the system evolves according to the dynamics dictated by the Hamiltonian. (At the annealing time scales used in this work, the dynamics is also influenced by environmental interactions.) Finally, at the end of each annealing process, the state of each (active) qubit is measured in the computation basis. Typically, this process is repeated many time, to produce sampled spin configurations.

The annealing parameter s lies in the range $[0, 1]$ and is a user-specified function of time. The functions $A(s)$ and $B(s)$ control the annealing and are in units of energy. While these two functions have qualitatively the same shape across various QPUs, they differ quantitatively (see Supplementary Information G). The user specifies the coupler values $\{J_{i,j}\}$ and the local fields $\{h_i\}$ to encode a particular Ising model on the hardware. Typical annealing experiments correspond to s ramping up linearly from 0 to 1, or $s = t/t_{\text{anneal}}$, with t denoting the annealing time.

Importantly, while the standard quantum annealing protocol requires s to start and end at 0 and 1 respectively, it is possible to "hold" or "pause" s at an intermediate value. We rely on this feature for all our experiments. As already explained in the paper, another important user-defined control is the function $g(t)$, which is known as the h-gain schedule. It controls the relative strength of the longitudinal field compared against the coupler strengths. By default, it is often set to $g(t) = 1$ throughout the annealing process. However, as described in main text and below, a time-varying h-gain schedule is the feature that allows our hysteresis protocols.

The objective of the simulation is to emulate, using a probabilistic sampling-based quantum computational approach, a magnetic field sweep protocol that can reveal collective magnetic memory in a transverse-field Ising model. The simulation is primarily controlled by two elements. The first is the transverse field in the Ising Hamiltonian, which drives state transitions quantum mechanically. (Note that due non-adiabaticity, along with thermal effects also cause transitions between different computational basis states.) The second is (a time-varying) longitudinal field which can be chosen to have different values at different spins. However, in all our simulations, we choose them to have the same value for all qubits. For our hysteresis experiments, we vary this function (as a linearly interpolated schedule) in a periodic manner, with each repetition starting initially at 0, then increasing it to the maximum positive value ($+H$), then to $-H$, followed by a return to $+H$. Here, the maximum applied H field is dependent on the D-Wave QPU hardware properties.

Importantly, the quantum annealers do not allow intermediate readout of qubit states in the middle of an anneal (when $\Gamma \neq 0$). Consequently, continuous monitoring of the magnetization of the Ising model can not be performed - instead, we must incrementally prepare slices of this longitudinal field protocol and then measure the states of the qubits at various intermediate point in the protocol. Additionally, because the sampling is probabilistic, for each schedule slice we perform multiple anneals and then average observable quantities are extracted for each parameter setting from the distribution of samples. The average magnetization M_z, which is a measure of the overall alignment of spins in the lattice, is the primary quantity that we use in order to demonstrate the magnetic hysteresis protocol. When reporting M_z, we reverse its sign in order to compensate for the sign of the D-Wave Hamiltonian of Eq. (2). At the end of each anneal-readout cycle, the states of all of the (active) qubits are measured in the computational basis (i.e., the Pauli z basis denoted by σ^z).

Figure S1 shows an example set of programmed schedules run on the D-Wave processor(s). These schedules are the two time-dependent control fields that facilitate the hysteresis simulation. This is effectively a two-sweep protocol (positive h-gain to negative h-gain, then negative h-gain to positive h-gain) that results in one closed hysteresis loop. During this changing h-gain field waveform, the system is paused at a fixed anneal fraction. The h-gain field is initialized at 0; changing the h-gain field from 0 to the maximum positive strength is required to initialize the two-sweep protocol, but we use this ramp to gradually polarize the state (as opposed to quenching this field rapidly to its maximum value). For this study, we use strictly the two-sweep technique shown in Figure S1 – but of course in principle one could use other sweep protocols. In practice we observe that the D-Wave quantum annealing hardware can frequently have remnant magnetization at initialization, which is why we use the initial longitudinal field ramp (shown by the red shaded region of Figure S1) to maximally polarize the system (up to the maximum longitudinal field we can apply on the hardware), before beginning the full sweep. Therefore, in practice we discard measurements during this initial ramp (red region), and only plot the closed hysteresis loop from the full sweep shown by the two blue regions. Some important specific components of the magnetic hysteresis simulation are defined, in detail, as follows:

Transverse field strength: The parameter s is the anneal schedule parameter that defines where in the standard anneal schedule the pause occurs, and is defined within the range $[0, 1]$. The term pause here refers to holding this hardware parameter s constant as a function of the simulation time - this is illustrated by the left

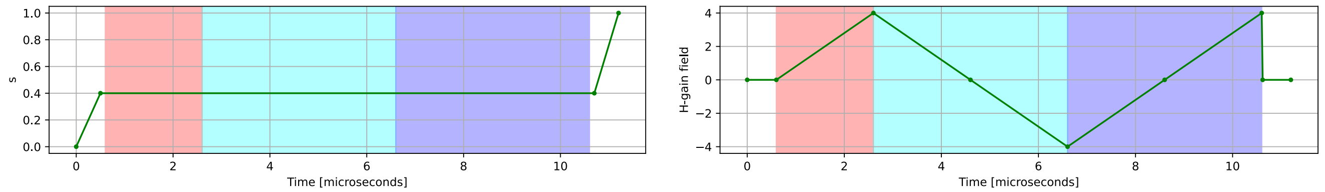

FIG. S1: The time-dependent quantum annealing hardware schedules that define the hysteresis simulation protocol. The anneal schedule that defines the proportion of the transverse field relative to the classical diagonal Hamiltonian energy scale is given by values of s specified at each point in time (left). The left-hand plot shows this anneal schedule for a specific example where the location of the anneal pause occurs at $s = 0.4$. The right hand plot shows the h-gain (longitudinal) field time dependent protocol, which is a uniform multiplier on all local fields. This is a sampling based protocol where measurements are made at many slices of this continuous time waveform (specifically, at slices at progressive time intervals within the three color shaded regions), and then averaged statistics of these measurements can be extracted. These two plots are showing the complete schedules at the very end of the simulation where the h-gain sweep is complete - intermediate simulations use shorter annealing times along with quenches to $h = 0$ before the qubit state measurements. Importantly, all non-zero h-gain fields are applied while the system is held at a specified transverse field proportion. The main regions of the simulation are marked as shaded vertical regions. Red shading shows the initial polarization ramp to get to the $+H$ polarized state; this is required to initialize the simulation since the hardware is initialized at $h = 0$ and can have remnant magnetization or noise at $h = 0$. Next, the cyan and blue shading show the two sweeps that implement one full (closed) hysteresis loop sweep, with the cyan going from $+H$ to $-H$ and the blue going from $-H$ to $+H$. We report the hysteresis data as the two sweeps that comprise the cyan and blue regions, neglecting the initial polarization ramp (red) as this is a initialization step.

D-Wave QPU Chip	Graph name	Qubits	Couplers	Maximum h-gain Field Strength	Avg. Node degree
`Advantage_system4.1`	Pegasus P_{16}	5627	40279	± 3	14.3
`Advantage_system6.4`	Pegasus P_{16}	5612	40088	± 4	14.2
`Advantage2_prototype2.6`	Zephyr $Z_{6,4}$	1248	10827	± 1.75	17.6

TABLE S1: Summary of the D-Wave quantum processing units (QPUs) used in this study. The average node degree refers to the average connectivity of the (undirected) hardware graph, where nodes represent qubits and edges represent couplers. Note that the h-gain field is defined in terms of device-specific normalized energy scales, meaning that the h-gain field units from one device are not exactly equal to the h-gain field strength on a different device.

sub-plot of Figure S1. Smaller s denotes stronger transverse field coupled with a weaker J, and larger s denotes weaker transverse field coupled with stronger J. This parameter we vary typically over the range of $s = 0.3$ to $s = 0.7$ so as to observe differences when state transitions are easier or harder. At very smaller and very large s values the hysteresis effect disappears. Importantly, this parameter s couples together the fields $A(s)$ (the transverse field) and $B(s)$ (the programmed Ising model), meaning we can not, for example, independently vary $A(s)$ while keeping $B(s)$ fixed. This is a hardware restriction of the current D-Wave quantum annealers, but this means that varying s is the only way to attenuate the transverse field strength. When reporting results, we typically report both this hardware defined normalized control parameter s along with the ratio between the transverse field (Γ) and J. The energy scale of the transverse field present in the simulation is very important. Namely, if there is not sufficient transverse field ($s = 1$), then there can be no state transitions and thereby no magnetization change. However, if there is only transverse field ($s = 0$), then there is no magnetic memory to be probed because the system does not change.

Programmable Local Fields: Whether the uniform local fields, for all active qubits, are set to all positive or all negative coefficients, the sign of these local fields should not significantly change the protocol - but could result in slight differences due to hardware noise or control errors. These local fields are what the h-gain time dependent control field acts on (as a multiplicative factor). In practice, in order apply the overall strongest longitudinal field possible (complemented by the time dependent multiplier of the h-gain field), we set this local field strength to the largest value that is allowed to be programmed on the hardware (which is 4 for these QPUs). Note that other patterns of local fields could be programmed on the D-Wave hardware, but generally we assume a uniform field will be applied with the goal of emulating the physical protocols that would be performed in a experimental hysteresis sweep on a real material. For all simulations we present in this study, we apply a uniform positive coefficient field. The use of the local field programmability (setting the h_i's), alongside the h-gain field, means that the Ising model that we probe with the hysteresis proto-

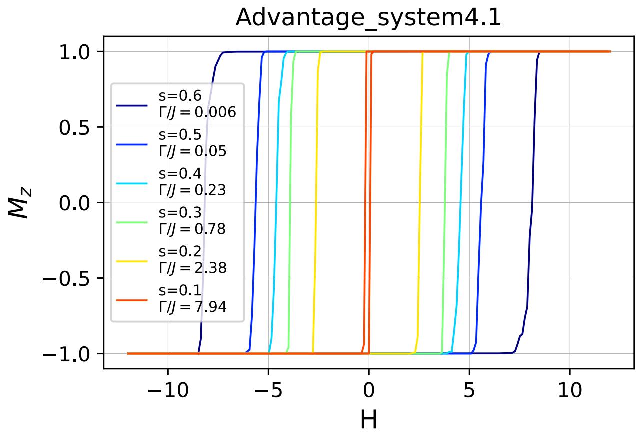

FIG. S2: Whole-lattice ferromagnetic model hysteresis run on the `Advantage_system4.1` processor. Magnetic hysteresis (magnetization M_z, normalized to theoretical saturation, vs. applied field H_z) of a hardware-graph ferromagnet run on `Advantage_system4.1` at various Γ/J values.

col must be defined entirely by the $J_{i,j}$ coupling terms.

Longitudinal Field Sweep Strength: This parameter is the strongest h-gain field (longitudinal field) that is applied during the sweep protocol This field is referred to as *h-gain* in the D-Wave hardware control parameters, but we will refer to this interchangeably as the *longitudinal field* or the *h-gain field*. This field is specified by the function $g(t)$ in Eq. (2). The applied field strength for both the positive and negative sign is always symmetric during the sweep (as illustrated by Figure S1-right). In principle, longitudinal fields that are weaker than the maximum allowable field that can be programmed on the hardware could be used, for example when studying minor loops. In practice, we use the strongest h-gain field that can be programmed on each D-Wave QPU. The (maximum) strength of the applied h-gain field is very important for this protocol, namely because ideally the simulation would reach full magnetic saturation – however if the longitudinal field is not strong enough relative to the magnetic system encoded on the hardware, then full saturation can not be reached (Table S1 lists the maximum longitudinal field that can be applied for each D-Wave device).

Longitudinal Field Points: As illustrated in Figure S1-right, the way that the h-gain schedule is defined is by a series of points which are then linearly interpolated between. At a minimum, these schedules used in this study require 7 points, but more complex schedules would need more h-gain schedule points (note that this is also true for more complex annealing schedules). The D-Wave QPU's used in this study allow only a maximum of 20 h-gain schedule points (and a maximum of 12 anneal schedule points) to be programmed by the user.

Anneal Schedule Ramps: How long the anneal schedule ramps take to reach the s anneal fraction, with the system having been initialized at $s = 0$ with maximum transverse field, and then similarly to go back to the $s = 1$ anneal fraction for qubit readout. The D-Wave hardware requires that the qubits are readout at $s = 1$ where there is no transverse field being applied. Ideally this change to $s = 1$ would be done nearly instantaneously, but in practice there is a constraint on how fast the anneal schedule can be changed meaning that these ramps do require up to 0.5 microseconds of simulation time. Although a required part of the simulation, we neglect these quenches in the hysteresis protocol in the sense that we do not measure at intermediate times during these quenches, and moreover we ensure that the total simulation times are larger than these quenches ensuring that a majority of the dynamics we observe are due to the pulsed longitudinal field. In practice, we use a ramp duration of 500 nanoseconds for all simulations both for the initial quench ramp and for the final readout ramp.

Longitudinal Field Ramp Decrease: How fast the h-gain field is reduced to 0 field during the intermediate slices of the protocol. Ideally this change would also be done nearly instantaneously. In practice, we set this duration to be 20 nanoseconds for all simulations to adhere to the maximum slope requirements on the D-Wave hardware for this control field. We want to set this field to zero during readout, and in particular during the hardware-required ramp immediately preceding readout, because otherwise the longitudinal field would continue to be applied during this ramp (e.g., a changing transverse field), instead of a static transverse field.

Turning Off the Longitudinal Field Pre-readout: This parameter defines how long we allow for the system to equilibrate after the initial anneal schedule ramp to the target pause at a given s value, and after the last step of the h-gain field is applied. We set this duration to be quite fast at 0.1 microseconds so as to minimize the effect of the transverse field while the longitudinal field is not applied. It is during this 0.1 microsecond anneal-schedule pause that the longitudinal field is turned off via a rapid quench of 20 nanoseconds (described above). The goal here of rapidly turning off the longitudinal field is to ensure that the driving anneal schedule, in particular the transverse field, is not changing while the longitudinal field is still on. Because of machine limitations however, both the h-gain field and the anneal schedule require finite times to turn them both off – ideally, both measurement of the qubits and turning off of the longitudinal field would occur effectively instantaneously. Longer equilibration times could be applied to this protocol, but this would result in the transverse field acting more on the system after the longitudinal field sweeps – in particular this causes more relaxation of the system towards a demagnetized state We note that simulations with longer equilibration times do exhibit magnetic hysteresis (at least with up to tens of microseconds of pause time), but for the simulations shown in this study we focus on very short equilibration times with the goal of attempting to readout the state of the qubits close to

the removal of the longitudinal field. For symmetry of the schedules, we also use this same duration of 0.1 microseconds of additional pause time, after the initial 0.5 microsecond quench to the target s, to wait before turning on the longitudinal field.

Total Simulation Time: The total length of the simulation can be changed quite significantly – the only requirement is that there is sufficient time to allow the h-gain sweep protocol to be implemented on the hardware and several points of that sweep to be sampled from. In D-Wave device parameter terminology this parameter is known as the annealing time, however in this case the process is not doing annealing, so we often interchangeably refer to this parameter as the simulation time. The minimum programmable annealing time on these D-Wave QPUs is 500 nanoseconds and the longest allowed annealing time is 2000 microseconds These QPUs do actually allow faster annealing times, down to 5 nanoseconds, but in that regime modified transverse field schedule control as well as longitudinal field control is not supported and therefore this protocol can not be applied in the anneal time regime of less than 500 nanoseconds. The short equilibration time and ramp durations combined have a simulation time of 1.2 microseconds. For all experiments reported in this study we use an annealing time of 11.2 microseconds. Note that this is the total annealing time at the very end of the simulation, intermediate steps of the simulation have a shorter total annealing time - for example the very first "slice" of the h-gain field sweep (post-maximum polarization) would use a total simulation time of ~ 3.2 microseconds.

Number of Sampled Points Along the Longitudinal Field Sweep: Because this protocol is a *sampling* based protocol, we need to select some number of points along the longitudinal field sweep to measure. For all simulations reported in this study, an annealing time of 11.2 microseconds is used, with ≈ 500 linearly spaced points along the longitudinal field sweep (this is 100 points along the five linear segments of the longitudinal field ramps in Figure S1, and a total of 400 points that form the closed hysteresis loops), and a total of 2000 (independent) anneal-readout cycles for each point along the hysteresis loop. $2,000$ samples for each point gives negligible finite sampling effects (e.g., shot noise) on the observables that we quantify such as M_z, but it is of course still present in these simulations – reducing shot noise more significantly could be an important consideration for simulations that are especially sensitive to the precision of measured observables. This number of linearly spaced points of ≈ 500 is motivated by the minimum annealing time resolution of 0.01 microseconds on D-Wave QPUs – for any other total simulation times that one could use in this protocol, the total number of sampled points should not cause the annealing time difference to drop below the minimum annealing time resolution of the hardware.

The Ising models used in this study are defined entirely by J couplings, and we use the maximum energy scale possible for all Ising model instantiations on the hardware; which is $+1$ and -1 (under the constraint that the ferromagnetic and antiferromagnetic couplers have symmetric energy scales) in terms of hardware-normalized programmable energy units. There are precision limitations on the hardware, inherently because of its analog nature, but the exact precision limits have not been rigorously quantified, and are dependent on the exact analog hardware specifications. Therefore, using the largest-coefficient coupling possible mitigates any analog precision error. All other programmable D-Wave hardware parameters not specified here are left at default values for the simulations that we report. Future studies could make use of statistic balancing calibrations of hardware parameters such as flux bias offsets so as to fine tune the simulations.

Supplementary Information B: Ferromagnetic Model Hysteresis on `Advantage_system4.1`

Figure S2 shows a complete set of hysteresis cycles on a hardware-graph defined ferromagnet, run on `Advantage_system4.1`. Compared with Figure 2, these hysteresis loops look nearly identical despite slight hardware differences.

The simulation of the fully ferromagnetic model defined on every coupler of the hardware graph of `Advantage2_prototype2.6` we do not show because the maximum possible longitudinal field strength that can be programmed on `Advantage2_prototype2.6` is weaker than the other two devices, which (combined with a slightly higher average qubit degree, see Table S1) resulted in the field being too weak to induce a magnetization reversal (meaning, the net magnetization, post initial polarization ramp, was constant).

Supplementary Information C: Averaged 2D Ising Model Magnetic Spin Structure Factor Heatmaps

We plot the magnetic spin structure factor (MSF) for momentum vector $\vec{q}$ computed at locations in a 200×200 uniformly spaced grid spanning $(-2\pi, 2\pi)$. The magnetic spin structure factor $S(\vec{q})$ can be computed for each spin configuration separately. When computing $S(\vec{q})$, we set that the lattice spacing is 1. For all our MSF plots, we compute and plot $|\langle S(q) \rangle|$ as a function of $\vec{q}$, where $\langle \circ \rangle$ denotes an averaging over 100 samples. This is motivated by averaging over a non-negligible number of samples to find if there are consistent correlations between spins during the hysteresis simulation. In particular, having too few samples causes the spin structure factor to suffer from finite sampling effects where there can be concentrations of the $S(q)$ vector by chance rather than showing a true underlying structure. We find 100 configurations is sufficient to average out finite sampling and finite system size effects. Magnetic structure factor allows us to visu-

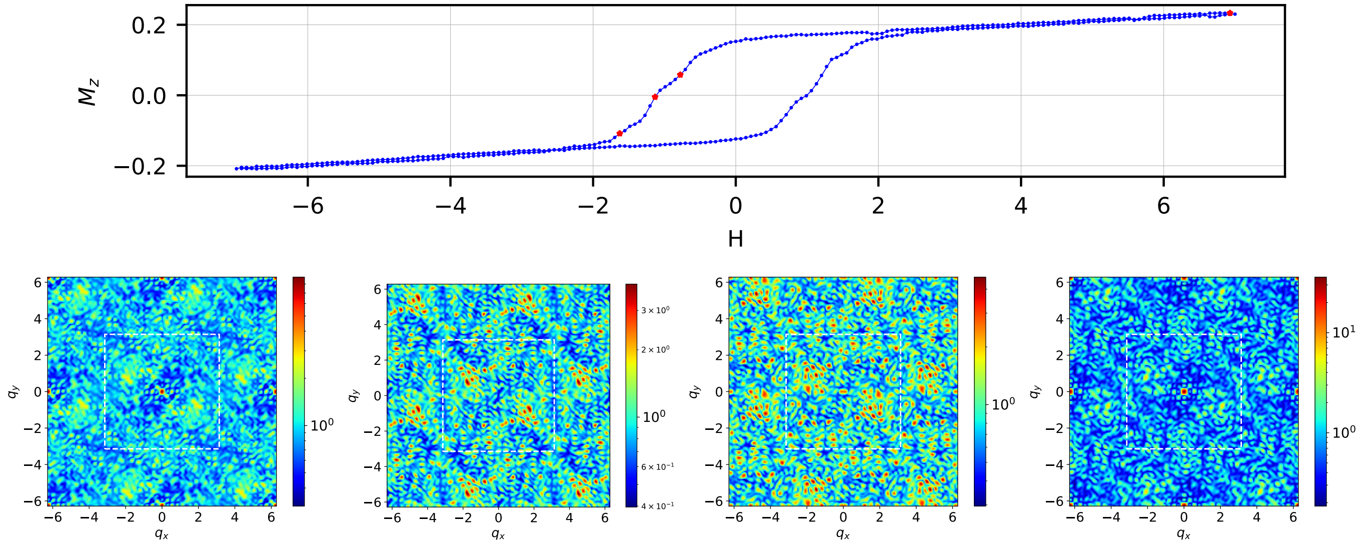

FIG. S3: Averaged magnetic structure factor plots extracted from a 2-dimensional disordered Ising model run on the `Advantage2_prototype2.6` processor at a higher ratio of Γ/J. Magnetic spin structure factor $|S(q)|$ heatmaps (bottom), averaged over the first 100 spin configurations sampled on the QPU, at representative points along the hysteresis cycle (top) for a 2D square grid (26 $\times$ 26 spins) $\pm J$ model run on the `Advantage2_prototype2.6` processor at $s = 0.3$. The order of the red points on the hysteresis loops correspond to the order of the MSF plots shown below the loop. Each MSF heatmap uses a scale determined by that $|S(q)|$ matrix, in other words the heatmap scale is not the same across the four heatmaps. The dashed white box outlines the first Brillouin zone.

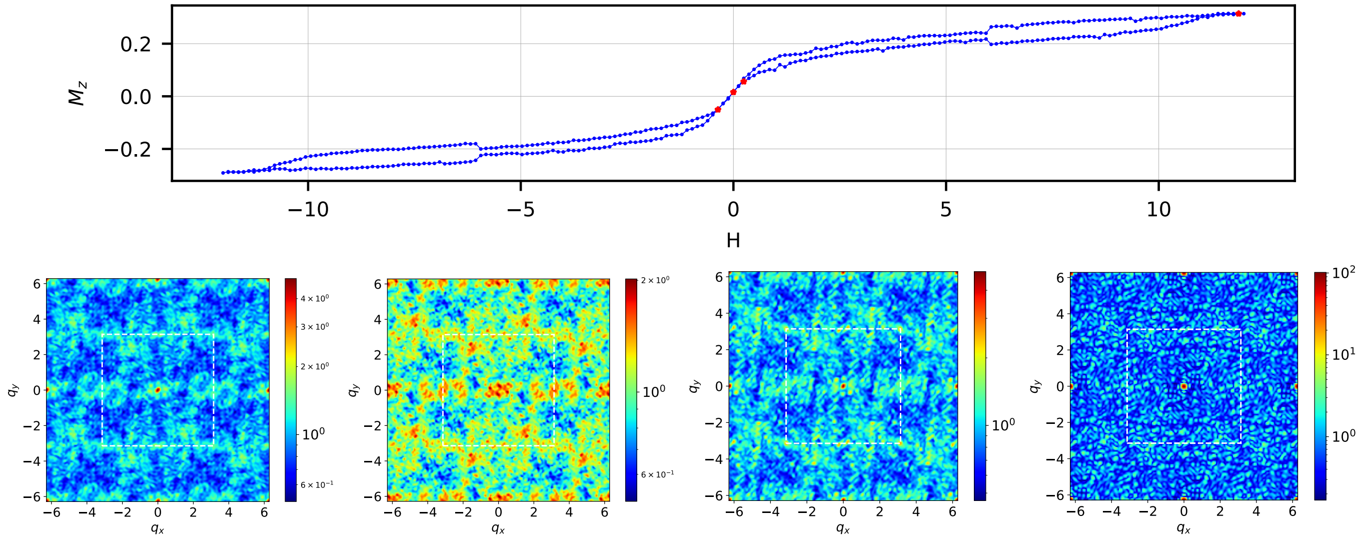

FIG. S4: Averaged magnetic structure factor plots extracted from a 2-dimensional disordered Ising model run on the `Advantage_system4.1` processor at a higher ratio of Γ/J. Magnetic spin structure factor $|S(q)|$ heatmaps (bottom), averaged over the first 100 spin configurations sampled on the QPU, at representative points along the hysteresis cycle (top) for a 2D square grid (32 $\times$ 32 spins) $\pm J$ model run on the `Advantage_system4.1` processor at $s = 0.3$. The order of the red points on the hysteresis loops correspond to the order of the MSF plots shown below the loop. Each MSF heatmap uses a scale determined by that $|S(q)|$ matrix, in other words the heatmap scale is not the same across the four heatmaps. The dashed white box outlines the first Brillouin zone.

ally examine the types of magnetic ordering that occur during these hysteresis cycles.

Figure S3 displays a hysteresis loop and selected magnetic spin structure factor plots, corresponding to specific points along the hysteresis cycle, for a $\pm J$ model on a 26 $\times$ 26 2D square lattice, implemented on `Advantage2_prototype2.6` for an anneal fraction of $s = 0.3$. As the maximum longitudinal field strength

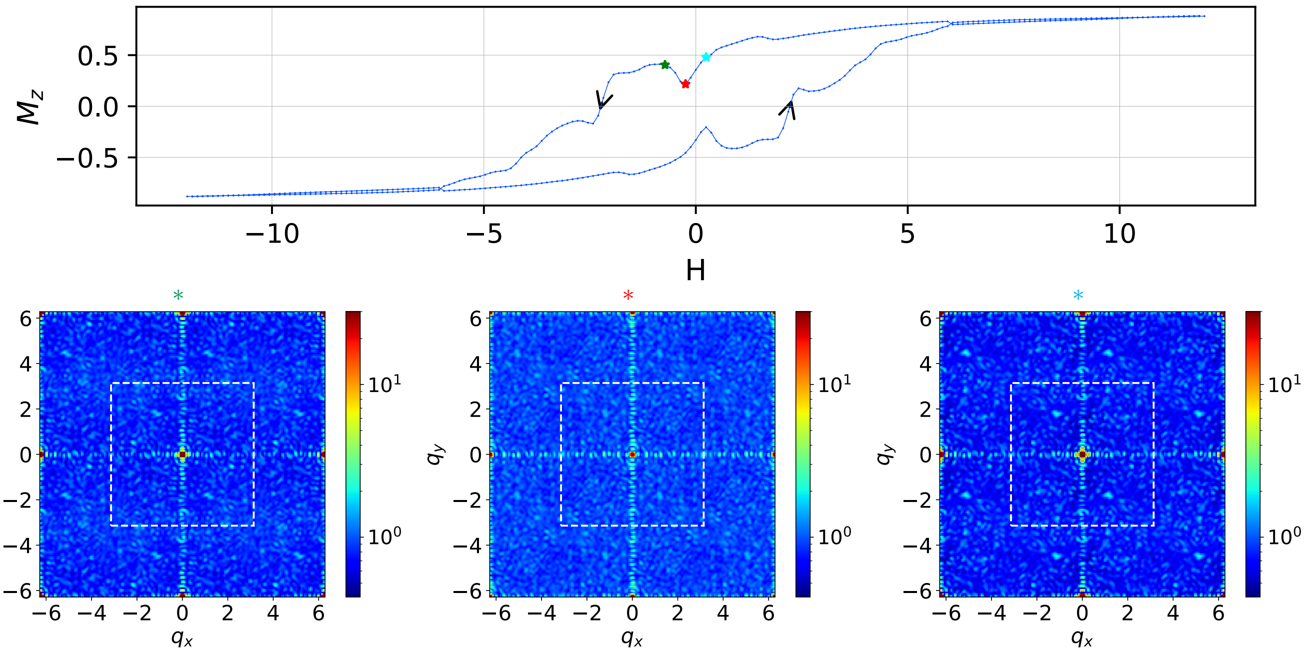

FIG. S5: Examination of averaged magnetic structure factors from a magnetic hysteresis cycle, on a 2-dimensional disordered model, that had a clear non-monotonic magnetization dip. This figure reports hysteresis data from a 32×32 $\pm J$ model run on `Advantage_system4.1` at $s = 0.6$ along with three MSFs (cyan point is before the dip, red point is at the dip, and green point is after the dip). The goal here is to probe the structure factor around and at a very clear non-monotonic magnetization dip. All MSF plots use a $|S(q)|$ heatmap on a log scale. The dashed white box outlines the first Brillouin zone. The three points are color-coded as green, red, cyan.

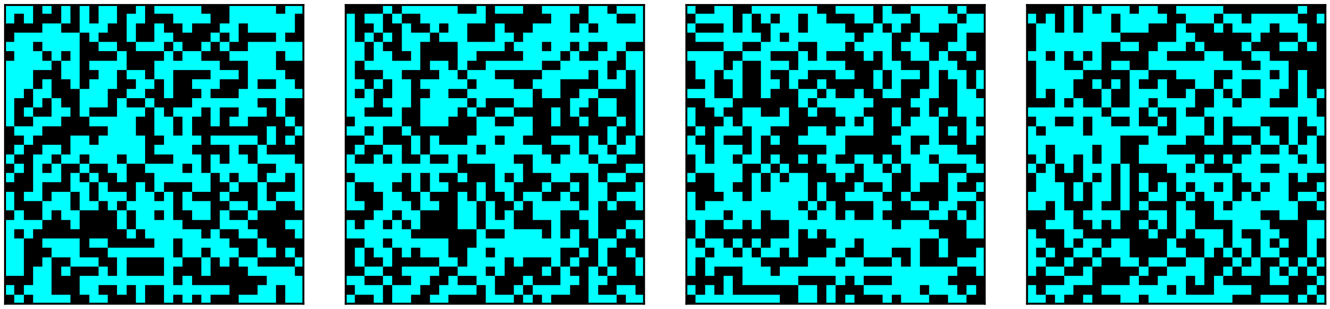

FIG. S6: Example measured spin configurations during hysteresis cycles run on a D-Wave QPU. Four representative example plots of individual samples (spin configurations) from the 32×32 $\pm J$ grid Ising model, generated on the `Advantage_system4.1` processor at $s = 0.7$, in the approximately de-magnetized region of the forward longitudinal sweep. In particular, these spin configurations come from an applied longitudinal field of -5 (in D-Wave hardware normalized units), with an average net magnetization M_z of ≈ 0.05 across these four individual samples. Cyan pixels denotes a spin up $+1$ and black pixels denotes a spin down -1.

is comparatively weak and the transverse field is strong at $s = 0.3$, we don't see magnetic saturation. Examining the MSF corresponding to the demagnetized regime (with MSF organized left to right corresponding to the points on the hysteresis loop left to right) we see weak peaks away from the center of the Brillouin zone indicating antiferromagnetic stripe ordering along a diagonal, breaking four fold rotational symmetry. As the system magnetizes, these peaks become more diffuse and a Bragg peak centered in the first Brillouin zone emerges under large applied field indicating the emergence of long range ferromagnetic ordering.

Figure S4 displays a hysteresis loop and selected MSFs for a $\pm J$ model on a 32×32 2D square lattice, implemented on `Advantage_system4.1` at an anneal fraction of $s = 0.3$. The strong transverse field in the $\pm J$ model prevents the system from saturating, and under small longitudinal field the remnant magnetization disappears. This can be understood by examining selected MSFs. Around the coercive field (near zero longitudinal field), the MSFs

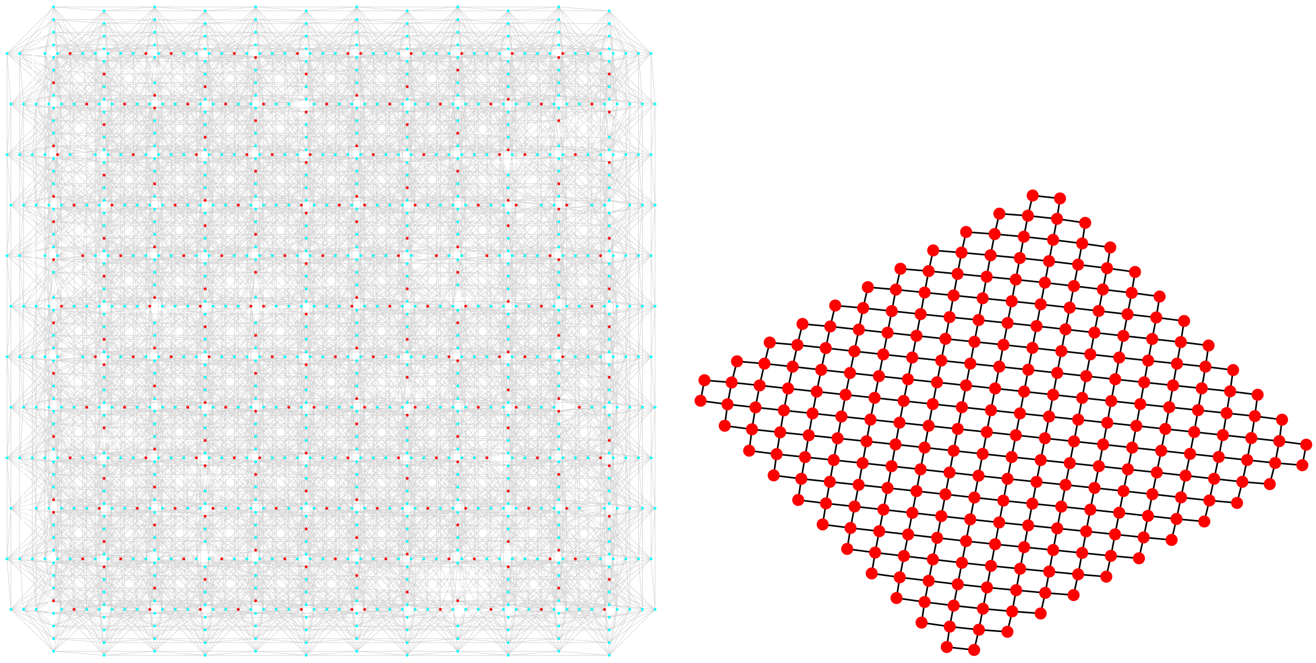

FIG. S7: Zephyr QPU hardware graph and a strictly planar subgraph extracted from the Zephyr graph. Planar subgraph of the `Advantage2_prototype2.6` Zephyr hardware graph. Left: the full hardware graph of the D-Wave QPU, where nodes are qubits and edges connecting them are hardware couplers. All couplers are shown as grey lines. Red denotes qubits that form the strictly planar 2D square grid subgraph, and cyan denotes all other qubits. Note that the hardware has couplers and qubits that are missing from the logical $Z_{6,4}$ graph. Right: the induced subgraph defined by the red qubits in the left hand hardware rendering. This subgraph is strictly a planar subgraph – there exist no other connections between these qubits besides those shown in the network rendering on the right. There are also other possible planar subgraphs of the hardware graph - here we use this single square grid embedding for all spin structure factor plots of the full hardware-defined spin glass models. The planar subgraph (right) contains exactly 264 nodes, whereas the hardware graph contains exactly 1248 nodes.

have a combination of both ferromagnetic ordering and antiferromagnetic ordering, indicated with diffuse peaks at both the center and the middle of the edges of the first Brillouin zones. As the system demagnetizes and the remnant magnetization drops to nearly zero the ferromagnetic and antiferromagnetic peaks become of nearly equal intensity. As the system magnetizes under strong longitudinal field we can see the appearance of strong ferromagnetic ordering with strong Bragg peaks centered in the first Brillouin zone with otherwise diffuse intensity away from these Bragg peaks.

Supplementary Information D: Magnetic Spin Structure Factor at a Non-Monotonic Dip

Figure S5 reports average magnetic spin structure factor plots at the three points (before, at, and after) the non-monotonic magnetization dip that was examined in Figure 5-c. These three MSF heatmaps were capped at $|S(q)| = 30$ for improved visualization clarity.

The magnetization reversal shown in Figure S5 is non-monotonic, resulting in a region of negative susceptibility shortly after the longitudinal field changes sign. Three corresponding spin structure factors are plotted, corresponding to the cyan, red and green points marked in the hysteresis loop. There is a train of sharp peaks along the primary axes of the magnetic spin structure factors due to the open boundary conditions of the square lattice. The correlation function $C(r)$ is obtained from the MSF principle axes and given in Figure 5-c. In reciprocal space, the one dimensional cuts along $k_x = 0$ and $k_y = 0$ produce a series of peaks separated by $\Delta k = 2\pi/32$, the envelope function of these peaks is the Fourier transform of $C(r)$. At the transient magnetization dip there is structural reordering. Before the dip there is a finite correlation length of $\xi \approx 1.7$, and the two-point correlation function $C(r)$ has an shoulder peak around $r = 7$, reflecting domain-wall structure size in unit cells. At the dip, we find that $\xi \approx 0.8$, i.e., correlations decay faster than just before and after the dip, and residual correlations beyond dominant short range correlations are spread widely demonstrating domains span a wide distribution of scales. Beyond this point, the magnetic configuration coarsens, and correlations and domain structures similar to before the dip return. The transient fragmentation of domains is apparent in the real space spin configurations of Figure 5-c. As the configurations are

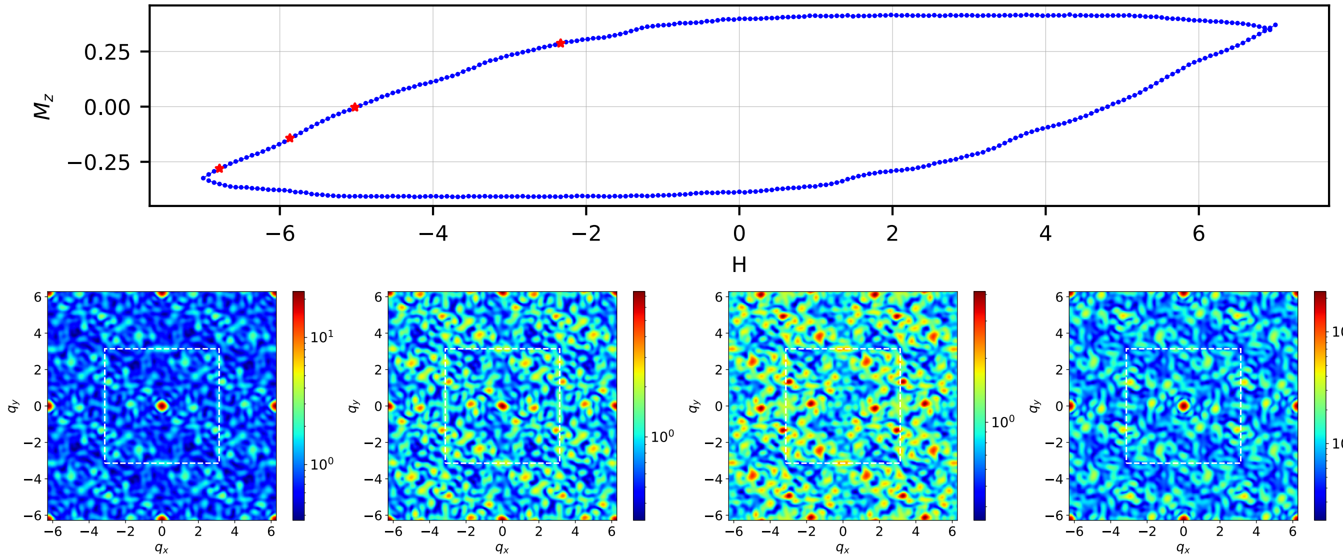

FIG. S8: Averaged magnetic spin structure factor from a 2-dimensional "slice" of the full Zephyr hardware graph defined $\pm J$, at various points during a hysteresis cycle run on the processor with a relatively weak Γ/J. Averaged magnetic spin structure factor $|S(q)|$, in log scale, from the quantum annealing hysteresis protocol at $s = 0.7$ (weaker transverse field, and stronger J) from a 264 node planar grid subgraph (e.g., 2D slice) of the Zephyr hardware graph `Advantage2_prototype2.6` defined $\pm J$ model at four specific points along the hysteresis cycle. The hysteresis curve on the top plot is the average single site magnetization M_z across the entire lattice, and the red asterisks denote specific points during the hysteresis cycle which we have extracted averaged MSFs of. The averaged MSF at each one of those points is given at the bottom - the order of these plots corresponds to the order of the red points on the hysteresis curve. Each MSF heatmap is averaged over the first 100 spin configurations measured on the quantum annealing hardware for progressively longer-duration longitudinal field slices. Here, the four MSF plots do not share the same heatmap scale. The dashed white box outlines the first Brillouin zone.

disordered, particularly at the dip, the peaks do not decay away from the central Bragg peak. When domains have a well defined characteristic scale the appearance of these structure peaks is suppressed. This transient negative susceptibility can be understood as a unique aspect of the TFIM and the $\pm J$ model, wherein when $H_z \approx 0$, the local environment, h^{internal}, experienced by each spin is reduced and the transverse field pushes the magnetization towards the paramagnetic regime. As the magnetization reversal continues, the system moves outside the regime dominated by the transverse field, the effective Z-basis Hamiltonian dominates again, and the still large-local effective field drives spins to align with the local magnetization, re-coarsening the magnetic configuration.

Supplementary Information E: 2D Spin Configuration Plots

Since we have full measurements of the spin configurations, we can also visualize the spin values for specific samples. Figure S6 renders four single spin configurations, in the form of a pixel grid, from one of the 32×32 2D $\pm J$ models, at a single fixed longitudinal field value in an approximately de-magnetized region of the hysteresis sweep. These real space configuration plots are intended to be examples of the type of spin orientation and ordering that we see during this demagnetized portion of the hysteresis sweep. Examining Figure S6 we see a lack of ordering and consistent domain size. Thus the demagnetized configurations are not well ordered but instead disordered and demagnetized.

Supplementary Information F: Strictly Planar 2D Square Grid Subgraph of the Zephyr Processor Graph for Magnetic Spin Structure Factor

Figure S7 shows a fixed 2D planar square grid subgraph induced from the hardware graph of the `Advantage2_prototype2.6` processor. This square grid can then be used to compute the MSF from a portion of the $\pm J$ model that is defined on the entire hardware graph. The key property that we need in order for this MSF to make sense is that the 2D slice of the somewhat densely interconnected hardware graph is strictly a planar subgraph meaning there no spurious interactions within this 2D grid, which is what Figure S7 shows us. Note that this planar subgraph is somewhat arbitrarily chosen - there exist other strictly planar subgraphs of the Zephyr hardware graph, here we choose just one as a demonstration of extracting a 2D slice view of the spin correlations during the hysteresis cycle. Figure S7 shows

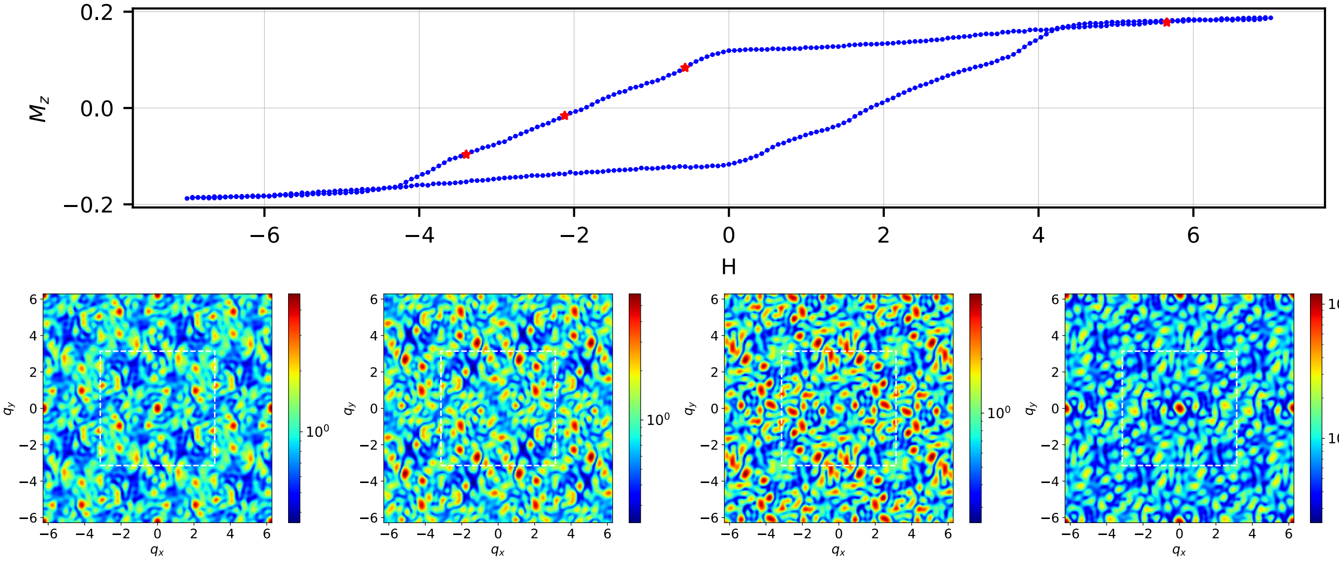

FIG. S9: Averaged magnetic spin structure factor from a 2-dimensional "slice" of the full Zephyr hardware graph defined $\pm J$, at various points during a hysteresis cycle run on the processor with a relatively strong Γ/J. Averaged magnetic spin structure factor $|S(q)|$, in log scale, from the quantum annealing hysteresis protocol at $s = 0.3$ (stronger transverse field, and weaker J) from a 264 node planar grid subgraph (e.g., 2D slice) of the Zephyr hardware graph `Advantage2_prototype2.6` defined $\pm J$ model at four specific points along the hysteresis cycle. The hysteresis curve on the top plot is the average single site magnetization M_z across the entire lattice, and the red asterisks denote specific points during the hysteresis cycle from which we have extracted averaged MSFs. The averaged MSF at each one of those points is given at the bottom - the order of these plots corresponds to the order of the red points on the hysteresis curve. Each MSF heatmap is averaged over the first 100 spin configurations measured on the quantum annealing hardware for progressively longer-duration longitudinal field slices. Here, the four MSF plots do not share the same heatmap scale. The dashed white box outlines the first Brillouin zone. These hysteresis simulations achieved a smaller maximum magnetization at the maximum applied longitudinal field compared to the weaker transverse field simulations of Figure S8.

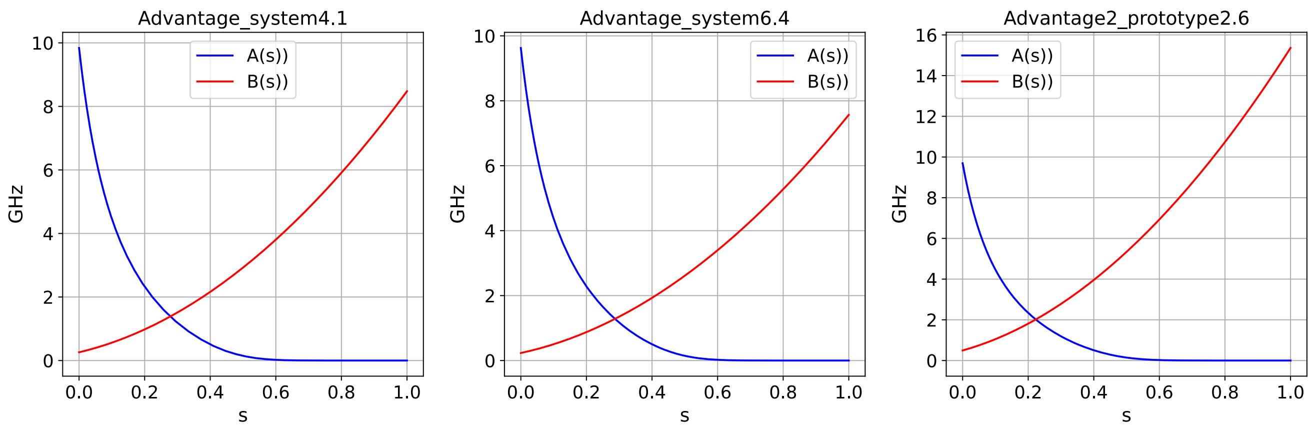

FIG. S10: Energy scale calibrations for all three D-Wave QPUs used in this study. These exact quantities are shown for reference because these energy scales determine the physical analog simulation performed within the hysteresis protocol.

this subgraph. Figures S8 and S9 show several averaged MSF heatmaps from this 2D slice of the full-hardware lattice defined $\pm J$ model on `Advantage2_prototype2.6`, during different points of the hysteresis cycles. These slices being 2D planar subgraphs makes them valid MSF correlations, however, they are still components of a reasonably complex hardware graph and therefore difficult to visually interpret beyond a few clear observations. For computing these planar subgraph MSFs, we use the same techniques and sample averaging described in Supplementary Information C.

Figure S8 displays a hysteresis loop along with four averaged MSFs along that magnetization curve for a 264 node planar grid subgraph (e.g., 2D slice) of the $\pm J$

model defined on the `Advantage2_prototype2.6` Zephyr hardware graph at $s = 0.7$. We observe the system demagnetize through the disappearance of the strong Bragg peak centered in the first Brillouin zone (far left MSF) and the appearance of peaks around the first Brillouin zone, most apparent in the third MSF. The zig-zag pattern of diffuse intensity and bright peaks is believed to be a result of selecting the planar subgraph from the full hardware graph. The planar subgraph, shown in Fig. S7 as red-dots in the full hardware graph, are a planar graph in an otherwise higher-dimensional connectivity graph. The number of spins in the full-hardware graph between neighboring spins in the planar sub-graph is not a fixed number, nor is it consistently even or odd, thus we are not guaranteed of an ordered planar sub-graph even in an ordered full-hardware graph. Thus the zig-zag structure in the demagnetized MSFs indicates that the full-hardware graph is demagnetized and we may be capturing higher order structure, such as stripe domains or a Néel order of which the planar subgraph is a two-dimensional slice.

Figure S9 displays hysteresis loop and example MSFs for the same 264 node 2D slice of the $\pm J$ model defined on the `Advantage2_prototype2.6` Zephyr hardware graph at $s = 0.3$. Restricting our analysis of the magnetic ordering to the planar sub-graph we can see the MSFs corresponding to a partially demagnetized system have antiferromagnetic and ferromagnetic ordering. Interestingly, the antiferromagnetic ordering corresponds to stripe ordering that persists in the partially magnetized system throughout the magnetization reversal. This corresponds to the ordering of antiferromagnetic peaks which form diagonal stripes across the MSFs. These stripe domains are weak ordering as the MSF have persistent fluctuations and diffuse intensity indicating a lack of global order. Under strong field the system gains stronger ferromagnetic ordering, indicated by the stronger Bragg peaks in the center of the first Brillouin zones, away from these peaks there is diffuse intensity indicating fluctuating antiferromagnetic order, particularly noticeable are signal intensity around the corner of the first Brillouin zone indicating Néel ordering.

Supplementary Information G: D-Wave QPU $A(s)$ and $B(s)$ functions

Figure S10 shows the exact energy scales of the D-Wave QPU control schedules $A(s)$ (Γ) and $B(s)$ (which denotes the energy scale of J). These are the quantities used in Eq. (2). The control parameter s is critical for setting the simulation properties of the magnetic hysteresis protocol, but the exact quantities are device specific and in particular using these calibrated device schedules one can compute the ratio Γ/J.

Supplementary Information H: Numerical Data Analysis Details

The magnetic spin structure factors are computed with the help of the Python 3 library Numba (45) to speed up the computations, as well as Networkx (46) and Numpy (47).

The area between the two sweeps of the magnetic hysteresis protocol are integrated numerically using two stages. First, is interpolation between all datapoints that are defined by averaged single-site magnetization M_z on the hysteresis curve as a function of the applied longitudinal field. The interpolation used is the PCHIP 1-D monotonic cubic interpolation (48) in scipy (49), using 10,000 points for the forward sweep and 10,000 points for the backward sweep. Then, the area between these curves is computed using the trapezoidal numerical integration rule in Numpy (47). All reported hysteresis areas are not normalized with respect the maximum possible area for each D-Wave device (meaning that the maximum possible areas for each device is different, determined by the hardware specifications).

REFERENCES

1. A. D. King, J. Raymond, T. Lanting, S. V. Isakov, M. Mohseni, G. Poulin-Lamarre, S. Ejtemaee, W. Bernoudy, I. Ozfidan, A. Y. Smirnov, M. Reis, F. Altomare, M. Babcock, C. Baron, A. J. Berkley, K. Boothby, P. I. Bunyk, H. Christiani, C. Enderud, B. Evert, R. Harris, E. Hoskinson, S. Huang, K. Jooya, A. Khodabandelou, N. Ladizinsky, R. Li, P. A. Lott, A. J. R. MacDonald, D. Marsden, G. Marsden, T. Medina, R. Molavi, R. Neufeld, M. Norouzpour, T. Oh, I. Pavlov, I. Perminov, T. Prescott, C. Rich, Y. Sato, B. Sheldan, G. Sterling, L. J. Swenson, N. Tsai, M. H. Volkmann, J. D. Whittaker, W. Wilkinson, J. Yao, H. Neven, J. P. Hilton, E. Ladizinsky, M. W. Johnson, M. H. Amin, Scaling advantage over path-integral Monte Carlo in quantum simulation of geometrically frustrated magnets. *Nat. Commun.* **12**, 1113 (2021).

2. P. Scholl, M. Schuler, H. J. Williams, A. A. Eberharter, D. Barredo, K. N. Schymik, V. Lienhard, L.-P. Henry, T. C. Lang, T. Lahaye, A. M. Läuchli, A. Browaeys, Quantum simulation of 2D antiferromagnets with hundreds of Rydberg atoms. *Nature* **595**, 233–238 (2021).

3. A. D. King, J. Carrasquilla, J. Raymond, I. Ozfidan, E. Andriyash, A. Berkley, M. Reis, T. Lanting, R. Harris, F. Altomare, K. Boothby, P. I. Bunyk, C. Enderud, A. Fréchette, E. Hoskinson, N. Ladizinsky, T. Oh, G. Poulin-Lamarre, C. Rich, Y. Sato, A. Y. Smirnov, L. J. Swenson, M. H. Volkmann, J. Whittaker, J. Yao, E. Ladizinsky, M. W. Johnson, J. Hilton, M. H. Amin, Observation of topological phenomena in a programmable lattice of 1,800 qubits. *Nature* **560**, 456–460 (2018).

4. P. Kairys, A. D. King, I. Ozfidan, K. Boothby, J. Raymond, A. Banerjee, T. S. Humble. Simulating the Shastry-Sutherland Ising model using quantum annealing. *PRX Quantum* **1**, 020320 (2020).

5. R. Harris, Y. Sato, A. J. Berkley, M. Reis, F. Altomare, M. H. Amin, K. Boothby, P. Bunyk, C. Deng, C. Enderud, S. Huang, E. Hoskinson, M. W. Johnson, E. Ladizinsky, N. Ladizinsky, T. Lanting, R. Li, T. Medina, R. Molavi, R. Neufeld, T. Oh, I. Pavlov, I. Perminov, G Poulin-Lamarre, C. Rich, A. Smirnov, L. Swenson, N. Tsai, M. Volkmann, J. Whittaker, J. Yao, Phase transitions in a programmable quantum spin glass simulator. *Science* **361**, 162–165 (2018).

6. A. D. King, C. Nisoli, E. D. Dahl, G. Poulin-Lamarre, A. Lopez-Bezanilla, Qubit spin ice. *Science* **373**, 576–580 (2021).

7. S. Ebadi, T. T. Wang, H. Levine, A. Keesling, G. Semeghini, A. Omran, D. Bluvstein, R. Samajdar, H. Pichler, W. W. Ho, S. Choi, S. Sachdev, M. Greiner, V. Vuletić, M. D. Lukin, Quantum phases of mat ter on a 256-atom programmable quantum simulator. *Nature* **595**, 227–232 (2021).

8. G. Semeghini, H. Levine, A. Keesling, S. Ebadi, T. T. Wang, D. Bluvstein, R. Verresen, H. Pichler, M. Kalinowski, R. Samajdar, A. Omran, S. Sachdev, A. Vishwanath, M. Greiner, V. Vuletić, M. D. Lukin, Probing topological spin liquids on a programmable quantum simulator. *Science* **374**, 1242–1247 (2021).

9. A. Lopez-Bezanilla, J. Raymond, K. Boothby, J. Carrasquilla, C. Nisoli, A. D. King, Kagomequbit ice. *Nat. Commun.* **14**, 1105 (2023).

10. T. Kadowaki, H. Nishimori, Quantum annealing in the transverse ising model. *Phys. Rev. E* **58**, 5355–5363 (1998).

11. S. Morita, H. Nishimori, Mathematical foundation of quantum annealing. *J. Math. Phys.* **49**, 125210 2008.

12. E. Farhi, J. Goldstone, S. Gutmann, M. Sipser, Quantum computation by adiabatic evolution. arXiv:quant-ph/0001106 (2000).

13. A. D. King, C. D. Batista, J. Raymond, T. Lanting, I. Ozfidan, G. Poulin-Lamarre, H. Zhang, M. H. Amin, Quantum annealing simulation of out-of-equilibrium magnetization in a spin-chain compound. *PRX Quantum* **2**, 030317 (2021).

14. G. E. Santoro, R. Martoňák, E. Tosatti, R. Car, Theory of quantum annealing of an Ising spin glass. *Science* **295**, 2427–2430 (2002).

15. A. Lopez-Bezanilla, A. D. King, C. Nisoli, A. Saxena, Quantum fluctuations drive nonmonotonic correlations in a qubit lattice. *Nat. Commun.* **15**, 589 (2024).

16. Y. Pei, C. Castelnovo, R. Moessner, Random transverse field effects on magnetic noise in spin systems. *Phys. Rev. B* **112**, 014416 (2025).

17. M. W. Johnson, M. H. S. Amin, S. Gildert, T. Lanting, F. Hamze, N. Dickson, R. Harris, A. J. Berkley, J. Johansson, P. Bunyk, E. M. Chapple, C. Enderud, J. P. Hilton, K. Karimi, E. Ladizinsky, N. Ladizinsky, T. Oh, I. Perminov, C. Rich, M. C. Thom, E. Tolkacheva, C. J. S. Truncik, S. Uchaikin, J. Wang, B. Wilson, G. Rose, Quantum annealing with manufactured spins. *Nature* **473**, 194–198 (2011).

18. T. Lanting, A. J. Przybysz, A. Y. Smirnov, F. M. Spedalieri, M. H. Amin, A. J. Berkley, R. Harris, F. Al tomare, S. Boixo, P. Bunyk, N. Dickson, C. Enderud, J. P. Hilton, E. Hoskinson, M. W. Johnson, E. Ladizinsky, N. Ladizinsky, R. Neufeld, T. Oh, I. Perminov, C. Rich, M. C. Thom, E. Tolkacheva, S. Uchaikin, A. B. Wilson, G. Rose, Entanglement in a quantum annealing processor. *Phys. Rev. X* **4**, 021041 (2014).

19. P. I. Bunyk, E. M. Hoskinson, M. W. Johnson, E. Tolkacheva, F. Altomare, A. J. Berkley, R. Harris, J. P. Hilton, T. Lanting, A. J. Przybysz, J. Whittaker, Architectural considerations in the design of a superconducting quantum annealing processor. *IEEE Trans. Appl. Supercond.* **24**, 1–10 (2014).

20. N. Dattani, S. Szalay, N. Chancellor, Pegasus: The second connectivity graph for large scale quantum annealing hardware. arXiv:1901.07636 [quant-ph] (2019).

21. K. Boothby, P. Bunyk, J. Raymond, A. Roy, Next-generation topology of D-Wave quantum processors. arXiv:2003.00133 [quant-ph] (2020).

22. K. Boothby, A. D. King, J. Raymond, “Zephyr topology of D-Wave quantum processors” (D-Wave Systems Inc., 2021); www.dwavequantum.com/media/2uznec4s/14-1056a-a_zephyr_topology_of_d-wave_quantum_processors.pdf.

23. P. G. De Gennes, Collective motions of hydrogen bonds. *Solid State Commun.* **1**, 132–137 (1963).

24. A. D. King, S. Suzuki, J. Raymond, A. Zucca, T. Lanting, F. Altomare, A. J. Berkley, S. Ejtemaee, E. Hoskinson, S. Huang, E. Ladizinsky, A. J. R. MacDonald, G. Marsden, T. Oh, G. Poulin-Lamarre, M. Reis, C. Rich, Y. Sato, J. D. Whittaker, J. Yao, R. Harris, D. A. Lidar, H. Nishimori, M. H. Amin, Coherent quantum annealing in a programmable 2,000 qubit Ising chain. *Nat. Phys.* **18**, 1324–1328 (2022).

25. A. D. King, A. Nocera, M. M. Rams, J. Dziarmaga, R. Wiersema, W. Bernoudy, J. Raymond, N. Kaushal, N. Heinsdorf, R. Harris, K. Boothby, F. Altomare, M. Asad, A. J. Berkley, M. Boschnak, K. Chern, H. Christiani, S. Cibere, J. Connor, M. H. Dehn, R. Deshpande, S. Ejtemaee, P. Farré, K. Hamer, E. Hoskinson, S. Huang, M. W. Johnson, S. Kortas, E. Ladizinsky, T. Lai, T. Lanting, R. Li, A. J. R. MacDonald, G. Marsden, C. C. Mc Geoch, R. Molavi, R. Neufeld, M. Norouzpour, T. Oh, J. Pasvolsky, P. Poitras, G. Poulin-Lamarre, T. Prescott, M. Reis, C. Rich, M. Samani, B. Sheldan, A. Smirnov, E. Sterpka, B. T. Clavera, N. Tsai, M. Volkmann, A. Whiticar, J. D. Whittaker, W. Wilkinson, J. Yao, T.J. Yi, A. W. Sandvik, G. Alvarez, R. G. Melko, J. Carrasquilla, M. Franz, M. H. Amin, Computational supremacy in quantum simulation. arXiv:2403.00910 [quant-ph] (2024).

26. A. D. King, J. Raymond, T. Lanting, R. Harris, A. Zucca, F. Altomare, A. J. Berkley, K. Boothby, S. Ejtemaee, C. Enderud, E. Hoskinson, S. Huang, E. Ladizinsky, A. J. R. MacDonald, G. Marsden, R. Molavi, T. Oh, G. Poulin-Lamarre, M. Reis, C. Rich, Y. Sato, N. Tsai, M. Volkmann, J. D. Whittaker, J. Yao, A. W. Sandvik, M. H. Amin, Quantum critical dynamics in a 5,000-qubit pro grammable spin glass. *Nature* **617**, 61–66 (2023).

27. J. Tindall, A. Mello, M. Fishman, M. Stoudenmire, D. Sels, Dynamics of disordered quantum systems with two- and three-dimensional tensor networks. arXiv:2503.05693 [quant-ph] (2025).

28. Y. Bando, Y. Susa, H. Oshiyama, N. Shibata, M. Ohzeki, F. J. Gómez-Ruiz, D. A. Lidar, A. del Campo, S. Suzuki, H. Nishimori, Probing the universality of topo logical defect formation in a quantum annealer: Kibble Zurek mechanism and beyond. *Phys. Rev. Res.* **2**, 033369 (2020).

29. M. H. Amin, Searching for quantum speedup in quasistatic quantum annealers. *Phys. Rev. A* **92**, 052323 (2015).

30. Z. Morrell, M. Vuffray, A. Y. Lokhov, A. Bärtschi, T. Albash, C. Coffrin, Signatures of open and noisy quantum systems in single-qubit quantum annealing. *Phys. Rev. Appl.* **19**, 0 3405 (2023).

31. J. Marshall, E. G. Rieffel, I. Hen, Ther malization, freeze-out, and noise: Deciphering experimental quantum annealers. *Phys. Rev. Appl.* **8**, 064025 (2017).

32. C. McCreesh, P. Prosser, J. Trimble, "The Glasgow Subgraph Solver: Using constraint program ming to tackle hard subgraph isomorphism problem variants," in *International Conference on Graph Transformation* (Springer, 2020), pp. 316–324.

33. Y. Ozeki, H. Nishimori. Phase diagram of the ±*J* Ising model in two dimensions. *J. Phys. Soc. Jpn.* **56**, 3265–3269 (1987).

34. G. Toulouse, "Theory of the frustration effect in spin glasses: I," in *Spin Glass Theory and Beyond: An Introduction to the Replica Method and Its Applications* (World Scientific Publishing, 1987), vol. 9, pp. 99–103.

35. H. Barkhausen, Zwei mit hilfe der neuen verstärker entdeckte erscheinungen. *Phys. Z* **20**, 401–403 (1919).

36. O. Perković, K. Dahmen, J. P. Sethna, Avalanches, Barkhausen noise, and plain old criticality. *Phys. Rev. Lett.* **75**, 4528–4531 (1995).

37. J. P. Sethna, K. A. Dahmen, C. R. Myers, Crackling noise. *Nature* **410**, 242–250 (2001).

38. E. Puppin, S. Ricci, L. Callegaro, Barkhausen jumps in a magnetic microstructure. *Appl. Phys. Lett.* **76**, 2418–2420 (2000).

39. L. Callegaro, E. Puppin, M. Zani, Barkhausen jumps and metastability. *J. Phys. D Appl. Phys.* **36**, 2036–2040 (2003).

40. J. Krempaský, G. Springholz, S. W. D'Souza, O. Caha, M. Gmitra, A. Ney, C. A. F. Vaz, C. Piamonteze, M. Fanciulli, D. Kriegner, J. A. Krieger, T. Prokscha, Z. Salman, J. Minár, J. H. Dil, Effcient magnetic switching in a correlated spin glass. *Nat. Commun.* **14**, 6127 (2023).

41. R. Yoshimi, K. Yasuda, A. Tsukazaki, K. S. Takahashi, M. Kawasaki, Y. Tokura, Current-driven magnetization switching in ferromagnetic bulk Rashba semiconductor (Ge,Mn)Te. *Sci. Adv.* **4**, eaat9989 (2018).

42. E. Östman, U. B. Arnalds, E. Melander, V. Kapaklis, G. K. Pálsson, A. Y. Saw, M. A. Verschuuren, F. Kronast, E. T. Papaioannou, C. S. Fadley, B. Hjörvarsson, Hysteresis-free switching between vortex and collinear magnetic states. *New J. Phys.* **16**, 053002 (2014).

43. J. A. Baldwin Jr., F. Milstein, Barkhausen noise power versus size of a minor hysteresis loop. *J. Appl. Phys.* **44**, 4739–4742 (1973).

44. S. Sabhapandit, D. Dhar, P. Shukla, Hysteresis in the random-field Ising model and bootstrap percolation. *Phys. Rev. Lett.* **88**, 197202 (2002).

45. S. K. Lam, A. Pitrou, S. Seibert, "Numba: A LLVM-based Python JIT compiler," in *Proceedings of the Second Workshop on the LLVM Compiler Infrastructure in HPC* (Association for Computing Machinery, 2015), pp. 1–6; https://doi.org/10.1145/2833157.2833162.

46. A. A. Hagberg, D. A. Schult, P. J. Swart, "Exploring network structure, dynamics, and function us ing networkx," in *Proceedings of the 7th Python in Science Conference*, G. Varoquaux, T. Vaught, J. Millman, Eds. (Los Alamos National Laboratory, 2008), pp. 11–15.

47. C. R. Harris, K. J. Millman, S. J. van der Walt, R. Gommers, P. Virtanen, D. Cournapeau, E. Wieser, J. Taylor, S. Berg, N. J. Smith, R. Kern, M. Picus, S. Hoyer, M. H. van Kerkwijk, M. Brett, A. Haldane, J. Fernández del Río, M. Wiebe, P. Peterson, P. Gérard-Marchant, K. Sheppard, T. Reddy, W. Weckesser, H. Abbasi, C. Gohlke, T. E. Oliphant, Array programming with NumPy. *Nature* **585**, 357–362 (2020).

48. F. N. Fritsch, J. Butland, A method for construct ing local monotone piecewise cubic interpolants. *SIAM J. Sci. Stat. Comput.* **5**, 300–304 (1984).

49. P. Virtanen, R. Gommers, T. E. Oliphant, M. Haberland, T. Reddy, D. Cournapeau, E. Burovski, P. Peterson, W. Weckesser, J. Bright, S. J. van der Walt, M. Brett, J. Wilson, K. J. Millman, N. Mayorov, A. R. J. Nelson, E. Jones, R. Kern, E. Larson, C. J. Carey, I. Polat, Y. Feng, E. W. Moore, J. Van Plas, D. Laxalde, J. Perktold, R. Cimrman, I. Henriksen, E. A. Quintero, C. R. Harris, A. M. Archibald, A. H. Ribeiro, F. P. dregosa, P. van Mulbregt, SciPy 1.0 Contributors, SciPy 1.0: Fundamental algorithms for scientific computing in Python. *Nat. Methods* **17**, 261–272 (2020).
